# Phosphoantigen/IL2 Expansion and Differentiation of Vγ2Vδ2 T Cells Increase Resistance to Tuberculosis in Nonhuman Primates

**DOI:** 10.1371/journal.ppat.1003501

**Published:** 2013-08-15

**Authors:** Crystal Y. Chen, Shuyu Yao, Dan Huang, Huiyong Wei, Helene Sicard, Gucheng Zeng, Hassan Jomaa, Michelle H. Larsen, William R. Jacobs, Richard Wang, Norman Letvin, Yun Shen, Liyou Qiu, Ling Shen, Zheng W. Chen

**Affiliations:** 1 Department of Microbiology and Immunology, Center for Primate Biomedical Research, University of Illinois College of Medicine, Chicago, Illinois, United States of America; 2 Herman B. Wells Center for Pediatric Research Indiana University, Indianapolis, Indiana, United States of America; 3 Innate Pharma, Marseille, France; 4 Institut für Klinische Chemie und Pathobiochemie, Justus-Liebig-Universität Giessen, Giessen, Germany; 5 Department of Microbiology and Immunology, Albert Einstein College of Medicine, Bronx, New York, New York, United States of America; 6 Harvard Medical School, Beth Israel Deaconess Medical Center, Boston, Massachusetts, United States of America; Portland VA Medical Center/Oregon Health and Science University, United States of America

## Abstract

Dominant Vγ2Vδ2 T-cell subset exist only in primates, and recognize phosphoantigen from selected pathogens including *M. tuberculosis*(Mtb). *In vivo* function of Vγ2Vδ2 T cells in tuberculosis remains unknown. We conducted mechanistic studies to determine whether earlier expansion/differentiation of Vγ2Vδ2 T cells during Mtb infection could increase immune resistance to tuberculosis in macaques. Phosphoantigen/IL-2 administration specifically induced major expansion and pulmonary trafficking/accumulation of phosphoantigen-specific Vγ2Vδ2 T cells, significantly reduced Mtb burdens and attenuated tuberculosis lesions in lung tissues compared to saline/BSA or IL-2 controls. Expanded Vγ2Vδ2 T cells differentiated into multifunctional effector subpopulations capable of producing anti-TB cytokines IFNγ, perforin and granulysin, and co-producing perforin/granulysin in lung tissue. Mechanistically, perforin/granulysin-producing Vγ2Vδ2 T cells limited intracellular Mtb growth, and macaque granulysin had Mtb-bactericidal effect, and inhibited intracellular Mtb in presence of perforin. Furthermore, phosphoantigen/IL2-expanded Vγ2Vδ2 T effector cells produced IL-12, and their expansion/differentiation led to enhanced pulmonary responses of peptide-specific CD4+/CD8+ Th1-like cells. These results provide first *in vivo* evidence implicating that early expansion/differentiation of Vγ2Vδ2 T effector cells during Mtb infection increases resistance to tuberculosis. Thus, data support a rationale for conducting further studies of the γδ T-cell-targeted treatment of established TB, which might ultimately help explore single or adjunctive phosphoantigen expansion of Vγ2Vδ2 T-cell subset as intervention of MDR-tuberculosis or HIV-related tuberculosis.

## Author Summary

Tuberculosis(TB), caused by *Mycobacterium tuberculosis*(Mtb), remains a leading cause of morbidity and mortality worldwide. While CD4+/CD8+ T cells are protective, role of γδ T cells in TB and other infections remains unknown in humans. Vγ2Vδ2 T cells exist only in primates, represent a dominant circulating γδ T-cell subpopulation, and recognize phosphoantigen from Mtb and some selected pathogens. Here, we determined whether earlier expansion/differentiation of Vγ2Vδ2 T cells during Mtb infection increased resistance to TB in macaques. Phosphoantigen plus IL-2 administration induced expansion and pulmonary accumulation of Vγ2Vδ2 T cells, significantly reduced Mtb counts and attenuated TB lesions in lung tissues. Expanded Vγ2Vδ2 T cells produced anti-TB cytokines IFNγ, perforin and granulysin, and co-produced perforin and granulysin in lung tissue. Perforin/granulysin-co-producing Vγ2Vδ2 T cells limited intracellular Mtb growth, and macaque granulysin killed Mtb bacteria, and inhibited intracellular Mtb in presence of perforin. Furthermore, expansion of Vγ2Vδ2 T effectors enhanced pulmonary responses of peptide-specific CD4+/CD8+ T cells, which correlated with the ability of Vγ2Vδ2 T effector cells to produce IL-12. These results provide first evidence implicating a protective role of Vγ2Vδ2 T effector cells in TB, supporting a rationale to explore Vγ2Vδ2 T-cell-targeted treatment of drug-resistant TB or HIV-related TB.

## Introduction

Tuberculosis(TB) remains one of the leading causes of morbidity and mortality worldwide among infectious diseases, and has become increasingly prevalent and deadly as a result of the emergence of multi-drug resistant (MDR) TB and HIV/AIDS pandemic [1], [2]. Given the possibility that TB patients usually have immune dysfunction, immune intervention regulating or enhancing anti-TB immune responses may be beneficial for clinical treatment of TB or MDR-TB. Although immune therapy or adjunctive immune plus antibiotics treatment has been long considered attractive for clinical treatment of TB, especially MDR TB, successful treatment modalities have not been identified due to the lack of an immune regimen that specifically targets immune components of anti-TB immunity. In fact, little is known about how human immune cells control a primary Mtb infection, despite that it is generally believed that human CD4+ T cells are important for resistance to TB [3], [4]. In-depth studies of Mtb-specific T effector cells may facilitate our understanding of anti-TB immune mechanisms and help identify potential immune intervention for drug-resistant TB.

Vγ2Vδ2 T cells exist only in primates, constitute 65–90% of total human circulating γδ T cells, and regulate or contribute to both innate and adaptive-like immune responses in infections [5], [6], [7], [8], [9]. Vγ2Vδ2 T cells recognize phosphoantigen (*E*)-4-hydroxy-3-methyl-but-2-enyl pyrophosphate (HMBPP) produced by *M. tuberculosis* (Mtb) or other selected pathogens in TCR-dependent fashion [10], [11], [12], [13]. Our decades-long studies in non-human primate models contribute to illustrating biology and immune responses of human Vγ2Vδ2 T cells in Mtb and other infections [6]. Recently, we and others have developed a unique manipulating system to remarkably expand Vγ2Vδ2 T cells *in vivo*. We showed that administration of phosphoantigen compound plus IL-2 induced remarkable expansion of Vγ2Vδ2 T cells, antagonized cytokine-induced Foxp3+ Treg, reversed Treg-mediated immune suppression, and attenuated pneumonic plague lesions in non-human primates [14], [15], [16], [17]. These findings provide a rationale for conducting a proof-of-concept study to determine whether phosphoantigen/IL-2 administration can expand/differentiate Vγ2Vδ2 T cells, improve immune system and resist TB in non-human primates. Finding may shed light on γδ T-cell-targeted immune intervention potential using a clinical drug zoledronate capable of expanding Vγ2Vδ2 T cells, as this drug is being tested as γδ T-cell-based therapeutic regimen for treatment of metastatic cancers [18], [19]. Studies of Vγ2Vδ2 T cell-targeted intervention can only be done in primates, as there is no evidence that γδ T cells from mice and other animals can recognize phosphoantigen and other microbial antigens [6].

## Results

### Picostim/IL-2 administration during pulmonary Mtb infection induced remarkable expansion of Vγ2Vδ2 T cells and pulmonary trafficking/accumulation of these cells

**Figure 1:**
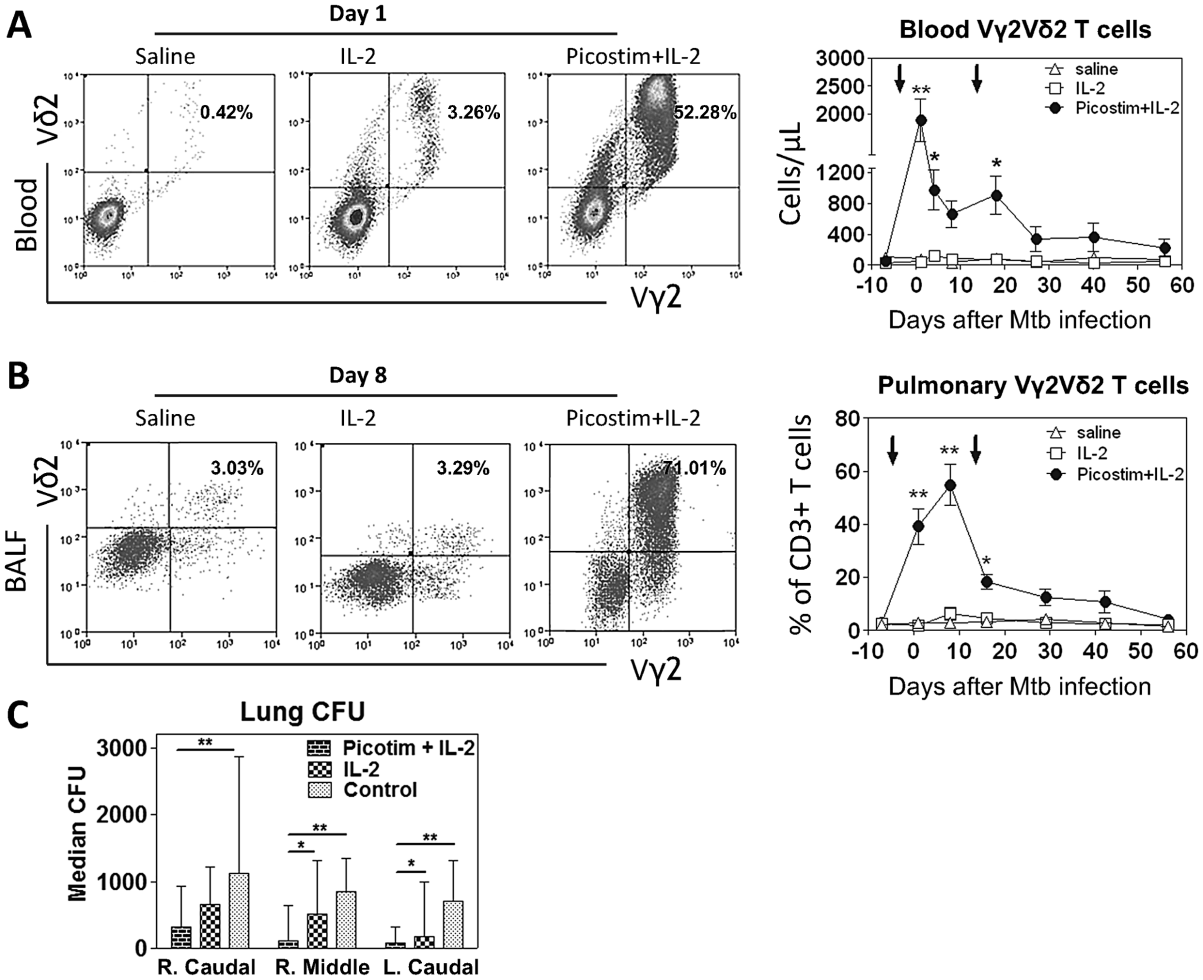
Intermittent Picostim/IL-2 administration during early pulmonary Mtb infection induced major expansion of Vγ2Vδ2 T cells and accumulation of them in pulmonary compartment, and led to reduction in Mtb bacterial burdens in lungs. (a) Representative flow histograms of percentages of Vγ2Vδ2 T cells gated on CD3(left) at day 1 postinfection (day 4 after 1^st^ treatment) and graph data (right) of absolute numbers of Vγ2Vδ2 T cells over time after treatments, respectively, in the blood. Percentages of Vγ2Vδ2 T cells in CD3 T cells are indicated in right upper quadruples. Graph data are means with SEM from nine macaques/per group. Vertical arrows denote the times for treatment. **p<0.01, *p<0.05, by both parametric and nonparametric analyses. (b) BAL fluid (BALF) data in a similar fashion as (a), except that flow data are on day 8 post infection(day 11 after treatments) and that numbers in graphs (right) are percentages of Vγ2Vδ2 T cells(n = 9 for each group). Increases in Vγ2Vδ2 T cells in BALF consist with accumulation of them in lung tissues of phosphoantigen/IL-2-treated macaques ([14] and data not shown). The data suggest changes in Vγ2Vδ2 T cells in the airway of the pulmonary compartment, not precisely in lung tissues. Please see Fig. S3c in Text S1 for immunohistocheminstry *in situ* analysis of γδ T cells in lung parenchyma and granulomatous tissues). (c) Mean CFU counts of bacilli in lung tissue homogenates of different lung lobes collected at the end point (at day 65) from Picostim/IL-2-treated and control groups. Lung tissue homogenates generated from each lobe was serially diluted and used for CFU enumeration(CFU/ml homogenates). **p<0.01, *p<0.05, respectively, by parametric and nonparametric comparisons between Picostim/IL-2-treated and control groups(n = 9/group). Data from control groups [22] were shown here again to prove the concept that phosphoantigen/IL2 expansion of Vγ2Vδ2 T cells can more apparently limit Mtb growth compared to saline/BSA and IL2 alone controls as all groups were evaluated simultaneously.

To conduct the proof-of-concept study, macaques were treated intermittently with the Picostim (identical to HMBPP except one carbon difference) plus IL-2 regimen [15] at day −3 and day 15, respectively, after pulmonary Mtb infection. Picostim/IL-2 administration during Mtb infection of macaques reproducibly induced remarkable expansion of Vγ2Vδ2 T cells (Fig. 1A). To minimize variation in evaluating infection/immunity in out-bred macaques, we performed 3 serial repeated experiments and, in each *in vivo* experiment, the test group and 2 control groups were simultaneously investigated. Vγ2Vδ2 T cells were expanded up to 60% from base-line 1% of total CD3+ T cells or increased to absolute mean numbers from ∼40/ul to ∼2000/ul after Picostim/IL-2 administration (Fig. 1A). Notably, expanded Vγ2Vδ2 T cells were able to traffic to and accumulate in the pulmonary compartment during Picostim/IL-2 treatment and Mtb infection (Fig. 1B). Virtually, such pulmonary accumulation of phosphoantigen-activated Vγ2Vδ2 T cells was consistent with increases in these γδ T cells in lung interstitial tissues and pulmonary lymphoid follicles[[14], and data not shown]. In contrast, control IL-2 alone or saline/BSA treatment did not induce significant increases in numbers of Vγ2Vδ2 T cells in the circulation and pulmonary compartments (Fig. 1a,b). The repeated Picostim/IL-2 treatment at day 15 after Mtb infection led to a subtle increase in the mean number of blood Vγ2Vδ2 T cells compared to the level at day 9, the time in which numbers of Vγ2Vδ2 T cells remained higher than baseline despite declining from the peak expansion following the initial treatment(Fig. 1a,1b). Thus, these results demonstrated that Picostim/IL-2 treatment during pulmonary Mtb infection of macaques induced major expansion of Vγ2Vδ2 T cells and pulmonary trafficking/accumulation of these cells.

### Picostim/IL2 administration, while expanding Vγ2Vδ2 T cells, could lead to reduction in Mtb bacterial burdens

Since Picostim/IL2 regimen induced major expansion and pulmonary trafficking/accumulation of Vγ2Vδ2 T cells, we examined whether this immune intervention could lead to changes in bacterial burdens after pulmonary Mtb infection. We measured bacterial CFU counts in lung tissue homogenates from right caudal lung lobe(infection site), right middle lobe and left caudal lobe at the end point after Mtb infection. The Picostim/IL2-treated group exhibited lower numbers of bacilli organisms in lung tissue homogenates from all studied lung lobes than the saline/BSA-treated and IL2-treated control groups, particularly in right middle and left caudal lung lobes than IL-2-treated control group (Fig. 1c). These findings showed that Picostim/IL-2 administration in early Mtb infection could expand Vγ2Vδ2 T cells and lead to reduction in Mtb bacterial burdens in lungs.

### Picostim/IL2 expansion of Vγ2Vδ2 T cells conferred immune resistance to TB lesions after pulmonary Mtb infection

**Figure 2:**
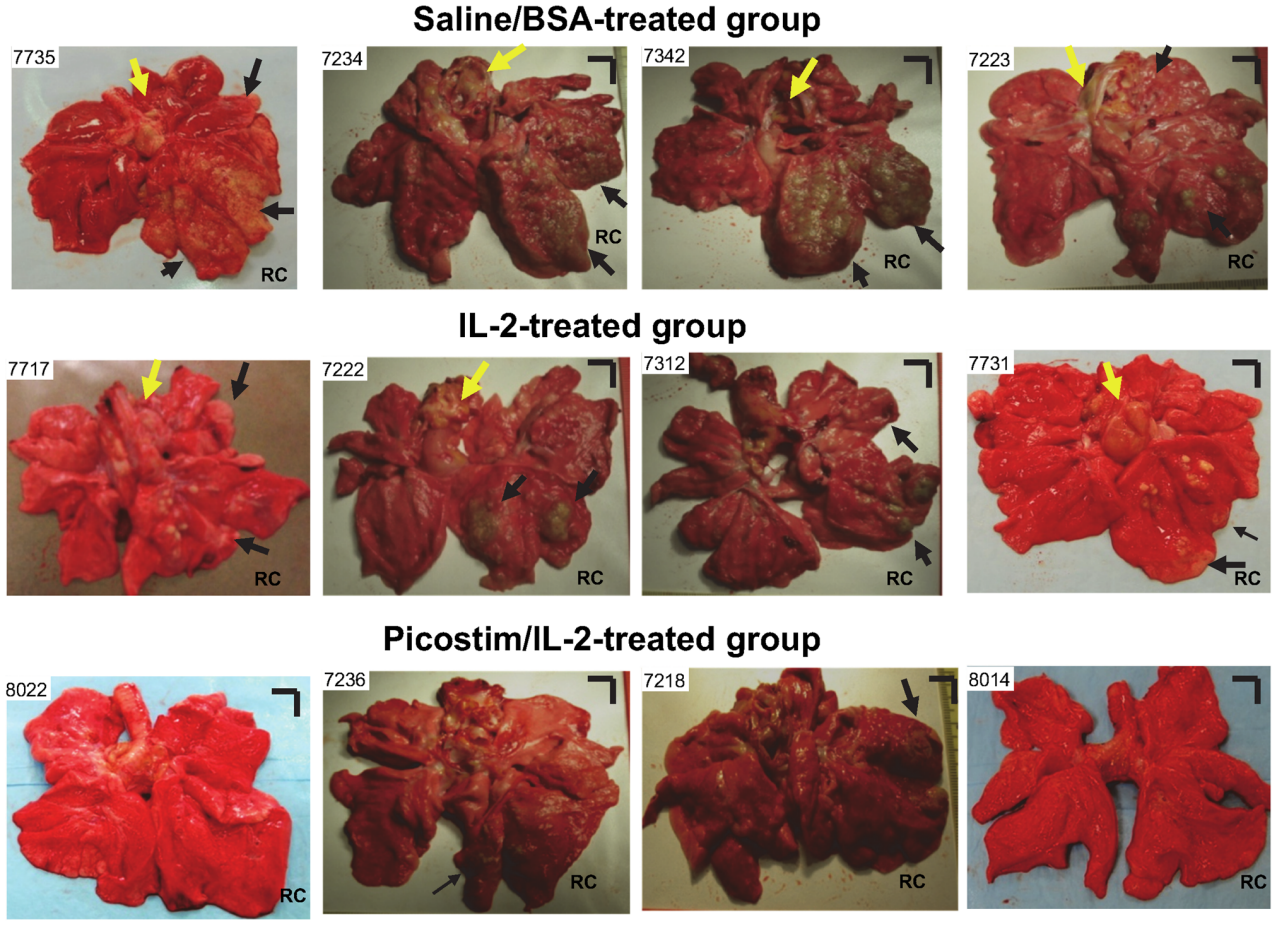
Picostim/IL2 treatment, while expanding Vγ2Vδ2 T cells, could confer immune resistance to TB lesions in lungs after pulmonary Mtb infection. Shown are digital photos of cut sections of lung lobes from 12 representatives of 27 macaques (9 for each group). Lungs and other organs were obtained in complete necropsy at day 65 after Mtb infection. Macaque ID numbers are shown in upper left corners, with the right caudal(RC) lobe (infection site) indicated in each photo. Extent and severity of the lesions could be adjudged based on the examples pointed by large arrows for caseation pneumonia or extensive coalescing granulomas and by small arrows for less coalescing or small granulomas. Yellow arrows indicate the enlarged hilar lymph nodes with caseation. Note that four Picostim/IL-2–treated macaques (ID 8022, 8014, 8013, 8006: first two animals shown in this figure and last two shown in Fig. S1 in Text S1) did not show any detectable gross TB lesions. Other five Picostim/IL-2–treated macaques displayed more focal, less coalescing or less caseating TB granulomas than control groups. (see Fig. S1 in Text S1). Vertical/horizontal bars at upper right corner of each photo represent the 1-cm scale derived from the fluorescence rulers of original photos. >50% of saline/BSA and 10% of IL-2–treated controls had extrathoracic TB dissemination, which was not seen in Picostim/IL-2-treated macaques. The efficacy evaluation was done sequentially in three separate trials each involving Picostim/IL-2 group and control groups of a total of 27 macaques.

**Figure 3:**
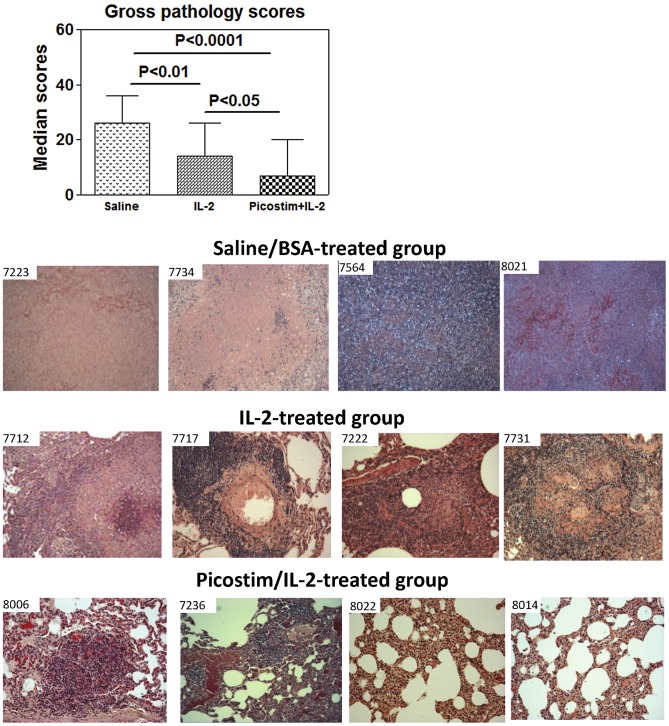
Picostim/IL2-treated group showed immune resistance to TB lesions in lungs compared to control groups. (a). Graph data of mean gross pathology scores for Picostim/IL-2-treated and control groups. The scores were calculated and compared as we previously described [21], [43]. n = 9/group. Control groups [22] were included here to prove the concept that phosphoantigen/IL2 expansion of Vγ2Vδ2 T cells can lead to increased resistance to TB. (b). Representative histopathology photos for Picostim/IL-2-treated and control groups. Also see Fig. S2 in Text S1 in the supplementary materials for histopathology from other macaques of three groups. Overall, saline/BSA-treated control macaques (represented in upper panel) exhibited widespread necrosis, fulminant TB pneumonia, and TB hemorrhage/thrombosis lesions. The alveolar septa are destroyed or indistinct. Most TB granulomas from IL2-treated macaques tended to be a lack of widespread necrosis, TB pneumonia, or TB hemorrhage in the sections from the right caudal lung lobe (infection site) and other lobes. In the Picostim/IL-2-treated group (lower panel), three macaques (ID: 8022,8014,8013) exhibited no detectable TB granulomas, four macaques predominantly showed well-contained TB granulomas with lymphocyte cuff, without widespread necrosis. Two of them exhibited histology similar to what were seen in IL-2-treated macaques. Original magnification ×100 for all photos. See Fig. S2 in Text S1 for similar contrasting data in other controls and IL-2–treated macaques.

We then sought to determine whether Picostim/IL2 manipulation of Vγ2Vδ2 T cells would increase resistance to TB lesions in lungs after Mtb infection. We conducted complete necropsy and gross pathology studies at day 65 after Mtb infection to examine detectable resistance to TB pathology and lesions in the Picostim/IL2-treated group in comparisons with the control groups. Interestingly, Picostim/IL2-treated macaques exhibited an increased resistance to TB lesions in lungs compared to saline/BSA and IL2-treated controls. Virtually, four animals (IDs:8022, 8014, 8013, 8006) in Picostim/IL2-treated group did not show any gross TB lesions in lung tissues (Fig. 2, Fig. S1 in Text S1), three animals displayed localized TB lesions limited to the right caudal lobe (infection site) of lungs (Fig. 2), and only two macaques showed apparent TB lesions involving the right caudal and middle lung lobes or left lower lobe (Fig. 2, Fig. S1 in Text S1). In contrast, all control macaques treated with saline/BSA exhibited severe/extensive caseating and miliary TB lesions or caseation pneumonia, and displayed extensive coalescing granulomas in right caudal/middle lobes and left lower lobe (Fig. 2, Fig. S1 in Text S1). TB lesions in these saline/BSA-treated macaques were readily disseminated to the opposite lung, hilar lymph nodes, and pleural or extrathoracic organs. All macaques except one in the IL2-treated control group developed TB lesions, and the lesions were more apparent than those in the Picostim/IL2-treated group(Fig. 2, Fig. S1 in Text S1). When the gross TB pathology or lesions was evaluated in details and compared between groups using the scoring system as we and others previously described [20], [21], [22], we found that the Picostim/IL2-treated group exhibited significantly milder TB lesions or pathology than saline/BSA-treated and IL2-treated control groups(Fig. 3a). Of note, the immune resistance to TB lesions after Picostim/IL2 treatment did not appear to be mediated by IL2 since Picostim/IL2-treated macaques showed less severe TB lesions or pathology than the IL2 alone group did (Fig. 3a). IL2 alone conferred detectable resistance to TB lesions compared to BSA/saline, but could not effectively reduce Mtb bacterial burdens([22], and Fig. 1c, Fig. 3a), whereas Picostim/IL2 expansion of Vγ2Vδ2 T effector cells decreased levels of Mtb infection(Fig. 1c).

Consistently, histopathology analyses also implicated immune resistance to TB in Picostim/IL2-treated macaques. TB granulomas in all saline/BSA-treated control macaques were apparently extensive, coalescing and widely necrotic in tissue sections from all studied lung lobes(Fig. 3b, Fig. S2 in Text S1). TB granulomas were seen in lung tissue sections from all IL2-treated macaques although these animals were less necrotic than saline/BSA-treated group (Fig. 3b, Fig. S2 in Text S1). Interestingly, among those four Picostim/IL2-treated macaques that did not show any gross lung TB lesions at necropsy, three macaques(IDs:8022, 8014,8013) did not exhibit any detectable microscopic TB granulomas in the lung tissue sections derived from right caudal and other lung lobes despite that we employed digital microscopic scanning for extensive analyses (Fig. 3b, Fig. S2 in Text S1). One of (ID:8006) those four animals showed only a few tiny, lymphocytic, non-necrotic microscopic granulomas(Fig. 3b). In other five Picostim/IL2-treated macaques, microscopic TB granulomas were smaller, more focal and much less necrotic than those in the saline/BSA-treated macaques, and similarly non-necrotic or more lymphocytic compared to IL2-treated macaques (Fig. 3b, Fig. S2 in Text S1).

Taken together, the gross pathology and histopathology results suggested that Picostim/IL2 expansion of Vγ2Vδ2 T cells could confer immune resistance to TB after pulmonary Mtb infection.

### Phosphoantigen/IL2-expanded Vγ2Vδ2 T cells differentiated to multi-functional effector subpopulations capable of producing anti-TB cytokines IFNγ, perforin and granulysin, and co-producing perforin/granulysin in lung tissues

**Figure 4:**
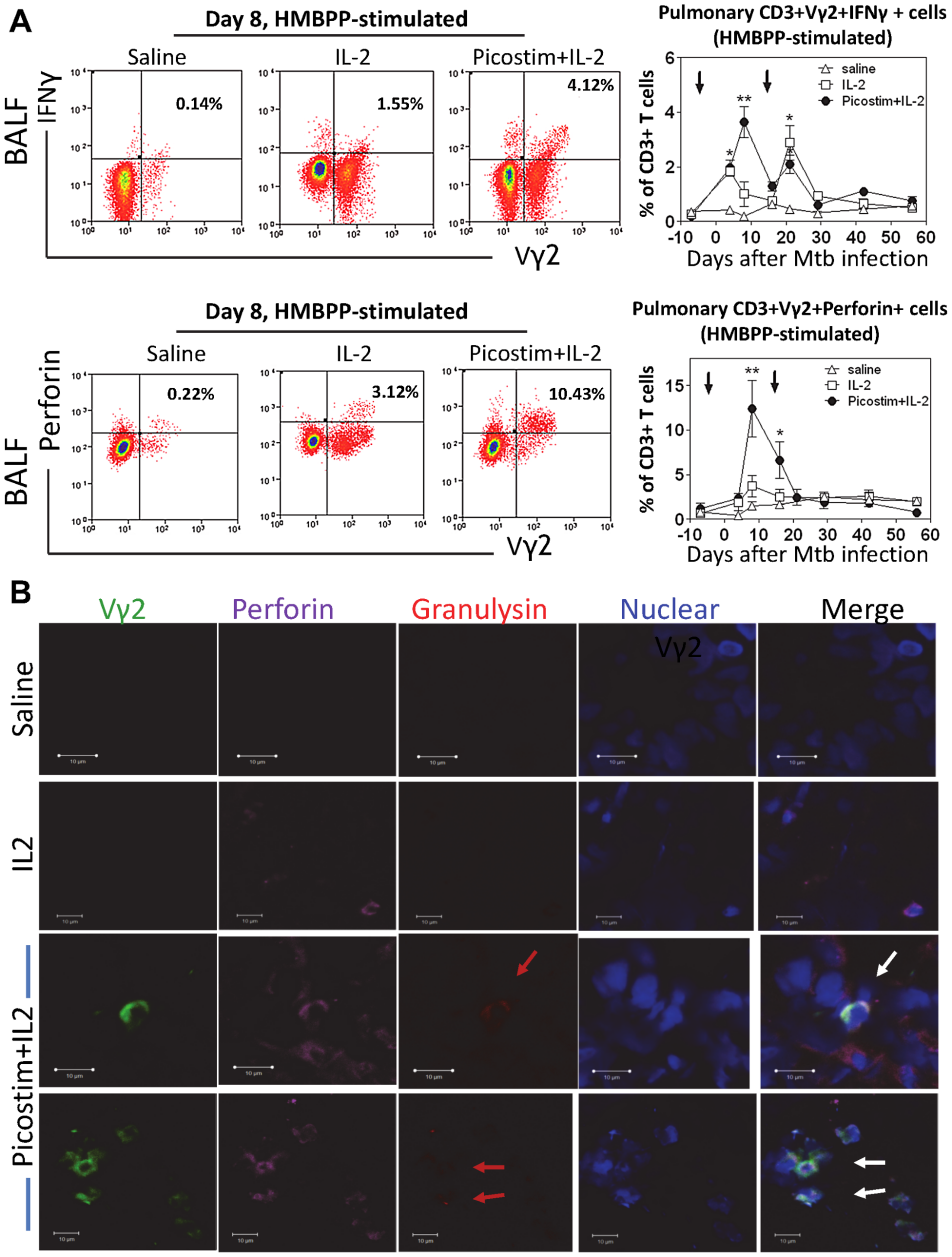
Phosphoantigen-expanded Vγ2Vδ2 T cells in pulmonary compartments differentiated and produced anti-Mtb cytokines IFNγ, perforin and granulysin, and co-produced perforin/granulysin in lung tissues. (a). Representative flow cytometry histograms of IFNγ-producing (upper panels) and perforin-producing Vγ2Vδ2 T effector cells gated on CD3 at day 8 postinfection and graph data (right) of numbers of Vγ2Vδ2 T effector cells in BALF collected overtime from Picostim/IL-2-treated and control groups. Effector cells were measured by ICS after HMBPP stimulation. n = 9/group for IFNγ effectors; n = 7/group for perforin effectors. * and ** denote p<0.05 and p<0.01, respectively, when analyzed by ANOVA or student t test. Effector cells measured by direct ICS without HMBPP stimulation was shown in Fig. S3a in Text S1. (b) Representative *in situ* confocal microscopic images (63× NA) of Vγ2Vδ2 T effector cells producing perforin and granulysin in lung tissue sections. The bottom two panels show that Vγ2 TCR (green) appear to co-localize with perforin (purple) and granulysin (red) in the merge images(co-localization marked by arrows) in lung tissue sections, suggesting that Vγ2 cells co-produce perforin and granulysin. Images at the top and middle panels show that no Vγ2 or granulysin was detectable in the lung tissue sections from representative macaques treated with saline or IL-2 alone, although perforin was detected in sections from IL-2-treated macaques. 10 um bars are indicated in images. All tissues sections were prepared from right middle and caudal lung lobes. Vγ2 cells were temporarily interpreted as Vγ2Vδ2 T cells as our previous studies demonstrated that Picostim or HMBPP/IL-2 exclusively expanded Vγ2Vδ2 T cells *in vivo* and most γδ T cells in lungs during Mtb infection were Vγ2Vδ2 T cells. Control isotype IgG did not give rise to any immune staining in the lung TB tissue sections (data not shown). See Sup Fig. 3b for additional confocal microscopic images of other macaques.

We then undertook mechanistic studies to examine if the immune resistance to TB coincided with anti-TB effector function of Vγ2Vδ2 T cells after Picostim/IL2 administration. Since mouse IFNγ and perforin are needed for anti-TB immunity [23], [24], [25], [26] and human granulysin kills Mtb [4], we examined if these anti-TB cytokines could be produced by Picostim-expanded Vγ2Vδ2 T cells in the pulmonary compartment. We employed both the conventional intracellular cytokine staining(ICS) upon *in vitro* antigen stimulation and the modified direct ICS assay without *in vitro* antigen stimulation since the direct ICS assay had been demonstrated to be useful for measuring T effector cells that were close to the *in vivo* setting during infections [22], [27], [28], [29], [30]. We found that appreciable numbers of Vγ2Vδ2 T cells in the pulmonary compartment were able to re-recognize HMBPP phosphoantigen and produce IFNγ over time after the Picostim/IL-2 administration(Fig. 4a). In fact, Picostim/IL2-expanded Vγ2Vδ2 T cells could produce IFNγ constitutively without the need for *in vitro* HMBPP phosphoantigen stimulation(Fig. S3a in Text S1). Similarly, pulmonary Vγ2Vδ2 T effector cells were able to produce perforin in the presence or absence of HMBPP stimulation, and these effectors peaked at ∼1 week in BAL cells after Mtb infection (Fig. 4a, Fig. S3a in Text S1). Furthermore, *in-situ* confocal imaging studies showed that Vγ2Vδ2 T effector cells producing perforin or granulysin were detectable in lung tissue sections derived from the Picostim/IL2-treated macaques (Fig. 4b, Fig. S3b in Text S1). Virtually, some of activated Vγ2Vδ2 T cells in lung tissues from Picostim/IL2-treated macaques were able to co-produce perforin and granulysin in situ (Fig. 4b, Fig. S3b in Text S1). In contrast, saline/BSA- or IL-2-treted control macaques did not show any perforin/granulysin-co-producing Vγ2Vδ2 T cells in lung tissues although perforin-producing cells (non-Vγ2) were detectable in IL-2-treated macaques(Fig. 4b). Immunohistochemistry analysis also demonstrated that Picostim/IL2-treated macaques exhibited more Vγ2 T cells in lung parenchyma and granulomas tissues than control IL2-treated and saline/BSA-treated animals even at the end time point(Fig. S3c in Text S1).

### Vγ2Vδ2 T effector cells producing granulysin/perform limited intracellular Mtb replication; macaque granulysin/perforin could inhibit Mtb growth

**Figure 5:**
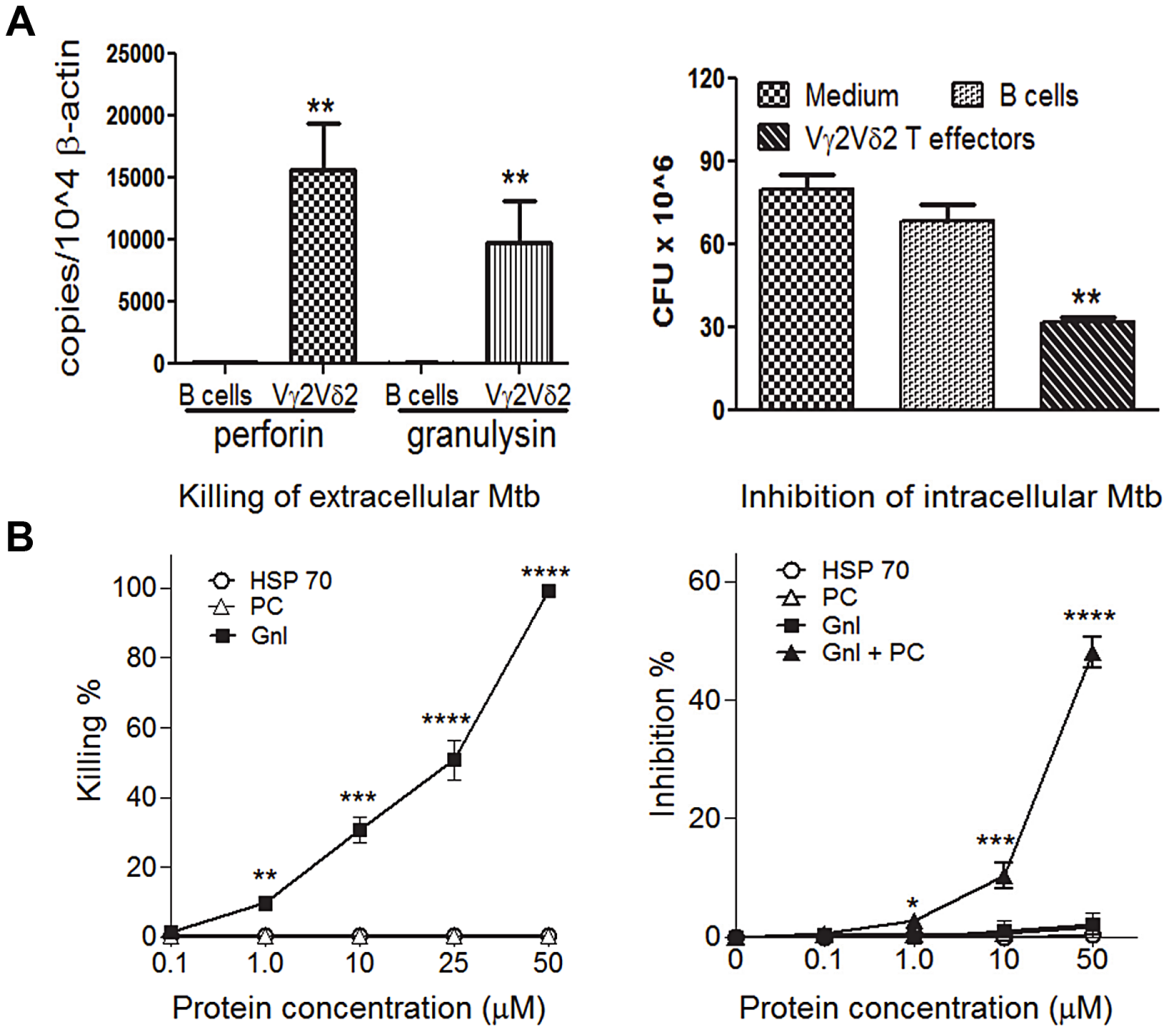
Vγ2Vδ2 T effector cells and macaque granulysin/perforin restricted Mtb growth. (a). Shown on left are real time quantitative PCR data for mRNA expression levels of perforin and granulysin expressed by macaque Vγ2Vδ2 T effector cells capable of restricting intracellular Mtb growth. The quantitation was done using the methods as we previously described [40]. Data are means with SEM from phosphoantigen-activated Vγ2Vδ2 T effector cells from 3 Mtb-infected macaques in 2 independent experiments. Control B cells were purified using CD20 mAb and immunemagnetic beads [29]. Right panel shows that co-culturing of Mtb-infected monocytes with Vγ2Vδ2 T effector cells expressing perforin/granulysin led to reduction in CFU bacterial counts. (b). Graph curves show that recombinant granulysin(Gnl), but not perforin(PC), killed extracellular Mtb (left); Gnl+PC inhibited intracellular Mtb growth(right). Data are the killing or inhibiting percentage of extracellular or intracellular Mtb CFUs relative to Mtb CFUs in medium-only control(see Methods). Data are means derived from Mtb-infected monocytes from three macaques in 3 experiments. ** p<0.001; ***p<0.0001; ****p<0.00001, by student t test.

Given that Picostim-differentiated Vγ2Vδ2 T cells produced anti-Mtb cytokines, we presumed that expanded Vγ2Vδ2 T effector cells could limit Mtb replication during Mtb infection. To test this hypothesis, we used the effector T cell-cloning technique as we previously described [31] to generate phosphoantigen-activated Vγ2Vδ2 T effector cells from PBL of Mtb-infected macaques [21] and assessed them for the ability to regulate intracellular Mtb replication. These Vγ2Vδ2 T effector cells expressed granulysin and perforin, and could limit Mtb growth in Mtb-infected monocytes (Fig. 5a). Consistently, some Vγ2Vδ2 T cells that expanded and differentiated *in vivo* at 14 days after Picostim/IL-2 treatment could recognize Mtb-infected autologous macrophages leading to inhibition of intracellular Mtb growth, and such inhibition could be reduced by antibodies against granulysin/perforin(FigS. 3d). The result indicated the need of granulysin and perforin secretion for anti-Mtb effector function and confirmed what was described in the *in vitro* study of human γδ T effector cells [32].

To examine a molecular mechanism whereby Picostim/IL2-expanded Vγ2Vδ2 T effector cells inhibited Mtb replication, we sought to determine whether macaque granulysin or perforin produced by Picostim-expanded Vγ2Vδ2 T effector cells exerted anti-Mtb activities. To this end, granulysin(Gnl) and active domain of perforin(PC) were expressed and purified using the *E. coli* expression system (Fig. S4 in Text S1, [33]), and then evaluated for the ability to affect extracellular and intracellular Mtb replication. We designed and produced active component of perforin [34], because the whole protein of macaque perforin was not optimally expressed and secreted in the *E. coli* expression system(data not shown). The active macaque granulysin killed extracellular Mtb at a concentration of 50 uM (Fig. 5b, left), and inhibited intracellular Mtb in monocytes/macrophages in the presence of active PC (Fig. 5b, right). The Fig. 5b data derived from the new system producing macaque granulysin/perforin help to explain anti-Mtb effector function of Vγ2Vδ2 T cells while confirming the result from the earlier study of human granulysin [35].

Taken together, these results consisted with the resistance to TB in Picostim/IL2-treated macaques, supporting the view that Vγ2Vδ2 T effector cells could limit intracellular Mtb replication during Mtb infection via production of granulysin and perforin.

### Picostim/IL2-expanded Vγ2Vδ2 T cells constitutively produced IL-12, and expansion of Vγ2Vδ2 T cells led to enhanced pulmonary immune responses of Mtb peptide-specific αβ CD4+ and CD8+ T effector cells

**Figure 6:**
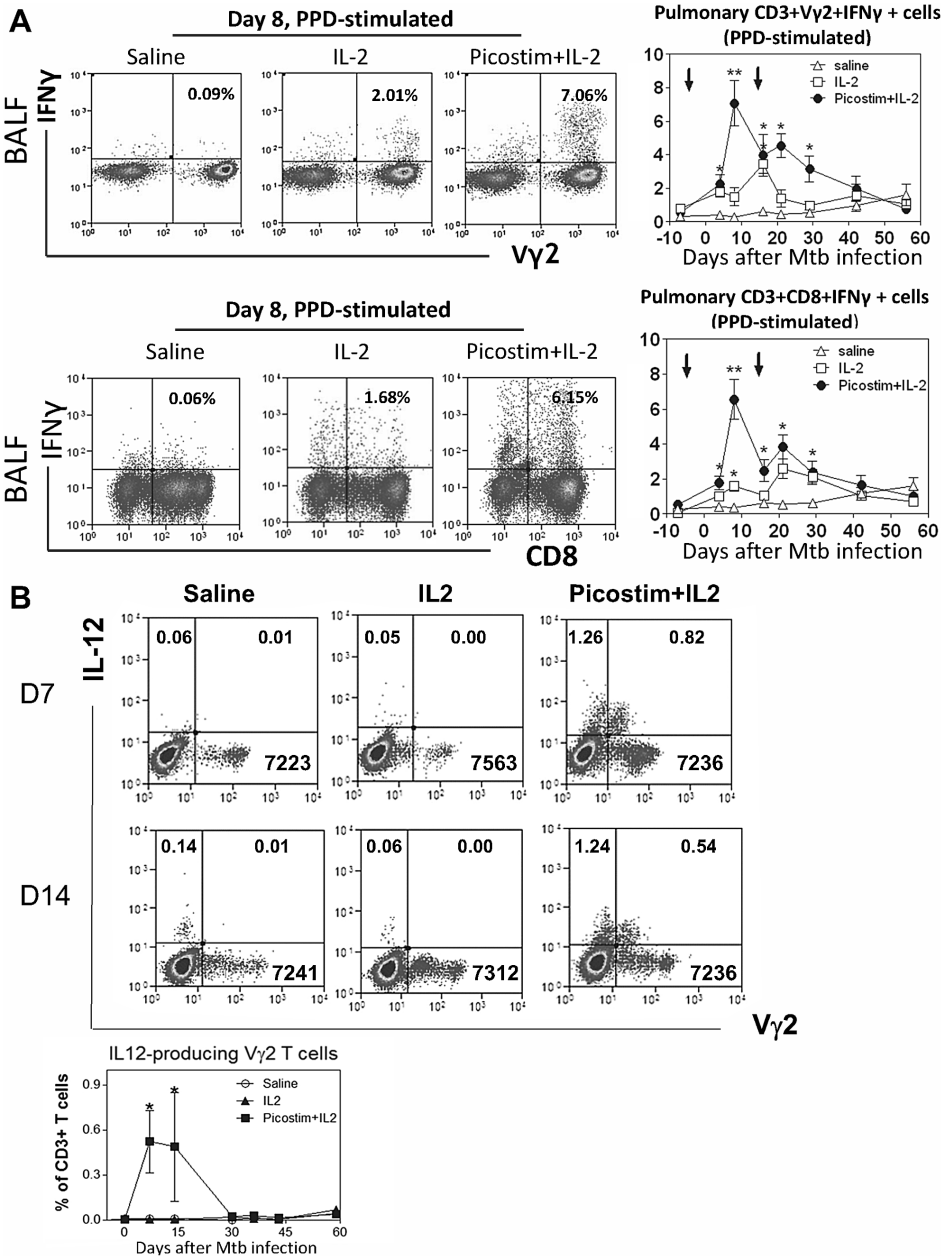
Picostim/IL2 expansion of Vγ2Vδ2 T cells led to enhanced pulmonary immune responses of Mtb peptide-specific αβ CD4+/CD8+ T effector cells, and Picostim/IL2-expanded Vγ2Vδ2 T cells *in vivo* could produce IL-12 *constitutive* without the need for *in vitro* stimulation(student t test used for p values). (a). Picostim/IL-2 treated macaques exhibited greater percentage numbers of PPD-specific IFNγ-producing CD4+ T cells (top) and CD8+ T cells(bottom) in BALF than saline/BSA-treated or IL-2-treated macaques. Effector cells were measured by ICS after PPD stimulation(n = 9/group). See Fig. S5 in Text S1 for T effector cells detected by direct ICS without antigen stimulation *in vitro*. (b). Representative cytometry histograms for Vγ2Vδ2 T cells producing IL-12 and graph data (bottom panel) for numbers of IL-12-producing Vγ2Vδ2 T cells from Picostim/IL-2, IL-2 alone or saline treatment of Mtb-infected macaques(n = 4/group). T effector cells producing IL-12 cytokine (clone C8.6, Miltenyi) were measured by the direct ICS method in the absence of antigen stimulation, analyzed by flow cytometry (see Methods), and expressed objectively as percents of total CD3+ T cells as cells were gated on CD3. Note that numbers in the upper left quadruple presumably denote percentages of IL12-producing CD3+ αβ+ T cells as they do not express γδ TCR.

Recent *in vitro* studies have shown that activated human Vγ2Vδ2 T cells can function as APC presenting peptide antigens to αβ T cells [36]. However, whether Vγ2Vδ2 T effector cells can enhance anti-microbial responses *in vivo* remains poorly characterized. As an additional mechanistic study, we investigated whether *in vivo* expansion of Vγ2Vδ2 T cells after Picostim/IL-2 treatment could augment immune responses of peptide Ag-specific αβ T cells in the context of immune resistance to Mtb infection. To this end, we compared frequencies of mycobacterium-specific CD4+ and CD8+ T effector cells producing anti-TB cytokine IFNγ between Picostim/IL2-treated and control groups of macaques since phosphoantigen was highly specific for Vγ2Vδ2 T cells, but not for CD4+ or CD8+ T cells. Picostim/IL2 expansion of Vγ2Vδ2 T cells did not lead to significant increases in frequencies of PPD-specific IFNγ-producing αβ T effector cells in the blood when compared to those from IL-2-treated group(data not shown). However, Picostim/IL2-treated macaques exhibited significantly greater numbers of Mtb-specific IFNγ-producing CD4+ T effector cells in the pulmonary compartment than control macaques treated with IL2 alone or saline/BSA during early phase of Mtb infection(Fig. 6a). Picostim/IL2 immune intervention also promoted greater increases in pulmonary PPD-specific IFNγ-producing CD8+ T effector cells than IL2 alone and saline control treatment(Fig. 6a). Moreover, an increased ability of CD4+/CD8+ T cells to constitutively produce IFNγ in the setting relevant to the *in vivo* condition was also seen in Picostim/IL2-treated macaques, as we detected an increased number of pulmonary CD4+ and CD8+ T effector cells capable of producing IFNγ without the need for peptide Ag stimulation *in vitro* (Fig. S5 in Text S1). We certainly realized that the pulmonary mobilization of these effector cells could not directly prove one of the immune mechanisms whereby expanded γδ T cells contribute to anti-TB immunity.

Since CD4+/CD8+ Th1 immune responses were often initiated and promoted by IL-12 production, we examined if Picostim-expanded Vγ2Vδ2 T effector cells were able to produce IL-12 leading to the enhanced pulmonary Th1 responses in Picostim/IL-2-treated macaques. Interestingly, we detected significant numbers of Vγ2Vδ2 T effector cells capable of constitutively producing IL-12 without the need for *in vitro* HMBPP stimulation after Picostim/IL2 administration, but not saline/BSA or IL2 alone treatment (Fig. 6b). These results suggested that Picostim/IL2-expanded Vγ2Vδ2 T cells were able to constitutively produce IL-12, and their expansion/differentiation led to enhanced pulmonary immune responses of Mtb peptide-specific αβ CD4+/CD8+ T effector cells.

## Discussion

Despite that human γδ T cells have been discovered for more than 25 years, the role of Mtb-specific Vγ2Vδ2 T cells in immunity against TB and other infectious diseases has not been defined in humans. Data from the current proof-of-concept study suggest that Picostim/IL-2 immune intervention during pulmonary Mtb infection can expand/differentiate Vγ2Vδ2 T effector cells, limit Mtb replication/dissemination, and attenuate TB lesions in non-human primates. Earlier negative finding for γδ T cells in mouse TB model does not appear to be conclusive [26], as there is no evidence that mouse γδ T cells can recognize Mtb phosphoantigen or other antigens [6]. Now we present the first *in vivo* evidence suggesting that phosphoantigen/IL2 expansion of Vγ2Vδ2 T cells can induce immune resistance to severe TB. This finding extends *in vitro* data demonstrating anti-Mtb effector function of human γδ T cells [8], [37], [38], [39], and appears to be novel and relevant to elucidating *in vivo* function of human Vγ2Vδ2 T cells.

Picostim/IL2 expansion/differentiation of Vγ2Vδ2 T effector cells mimics the natural immune stimulation driven by Mtb phosphoantigen and IL2 in Mtb infection. The Picostim/IL2 intervention simply moves the selective expansion/differentiation of Vγ2Vδ2 T effector cells earlier and to a greater extent, since production of phospohoantigen and IL2 will become prominent only in a later phase of Mtb infection. In fact, Mtb infection or BCG vaccination can induce remarkable expansion of Vγ2Vδ2 T cells at ∼3–4 weeks postinfection, and rapid recall expansion of Vγ2Vδ2 T cells in BCG-vaccinated macaques correlates with the vaccine-induced protection against life-threatening TB [9]. Earlier and greater-magnitude expansion of Vγ2Vδ2 T cells during Mtb infection appears to create a proof-of-concept setting in which to examine immune function of Mtb-specific Vγ2Vδ2 T effector cells in primates, as rapid clonal expansion and pulmonary trafficking of Mtb-specific CD4+/CD8+ T cells have been interpreted as important immune events in immunity against TB in macaques [40]. The resistance to TB after phosphoantigen treatment could be due to killing and blunting of Mtb as they enter the pulmonary compartment after infection, since the treatment is given during early Mtb infection rather than established TB disease.

It should be pointed out that only some of expanded Vγ2Vδ2 T cell subpopulations are protective although almost all γδ T cells expressing Vγ2Vδ2 TCR have been shown to recognize Mtb phosphoantigen [6], [10], [39]. Consistent with this concept, we and others including Spencer/Hoft et al have repeatedly shown that only certain percentages of expanded Vγ2Vδ2 T cells can differentiate into effector cells capable of producing anti-TB cytokines or inhibiting mycobacterial growth during phosphoantigen/IL2 treatment or Mtb infection. It is also noteworthy that phosphoantigen/IL2 treatment of chronic TB may not be as efficacious as the treatment of early-phase or primary TB, since patients with chronic TB often exhibits anergy or insufficiency of γδ T cell effector function [6]. In addition, an *in vitro* study by Spencer/Hoft et al showed that large numbers of phosphoantigen-activated γδ T cells could not recognize BCG-infected target cells [39].

The immune resistance to Mtb infection and TB appears to result from multi-effector functions of phosphoantigen-expanded Vγ2Vδ2 T cells rather than IL-2 immune activation since Picostim/IL2-treated macaques exhibited lower Mtb CFU counts and milder TB lesions in most lung lobes than IL2-treated controls. It is noteworthy that IL-2 treatment confers resistance to TB lesions compared to BSA/saline treatment [22]. Notably, IL-2 administration predominantly expanded CD4+CD25+Foxp3+ Treg [15], whereas Picostim/IL-2 regimen massively expanded Vγ2Vδ2 T cells in mycobacterial infection, down-regulated IL2-induced expansion of IL2-induced Treg cells and reversed Treg-associated immune suppression *in vivo* [15]. Although IL2 alone is protective against TB lesions, the regimen could not effectively reduce Mtb bacterial burdens([22], Fig. 1c, Fig. 3a). Picostim/IL2 expansion of Vγ2Vδ2 T cells led to a decreased level of Mtb infection and more apparent resistance to TB than IL-2 expansion of Treg and T effectors([22], Fig. 1c, Fig. 3a). Of note, all IL2-treated macaques except one developed TB lesions. In contrast, approximately 45% of phosphoantigen/IL2-treated macaques did not exhibit detectable TB, and gross pathology scores in phosphoantigen/IL2-treated group are significantly smaller than those in IL2-treated and saline/BSA-treated control groups. While anti-Mtb effector function of phosphoantigen-expanded Vγ2Vδ2 T cells appears to be relevant to controlling Mtb infection and attenuating TB, the ability of Vγ2Vδ2 T effectors to mediate homeostasis against inflammation and tissue damage may also contribute to the immune resistance to TB. This notion is supported by our recent observation that phosphoantigen/IL2-expanded Vγ2Vδ2 T effector cells can produce a homeostatic mediator FGF-7, and coincide with attenuation against pneumonic plague damages of lung tissues after pulmonary infection even with extracellular *Y. pestis* [14], whose protection often requires neutralizing Ab.

Data from the mechanistic experiments suggest that phosphoantigen/IL2-expanded Vγ2Vδ2 T effector cells producing granulysin/perforin during Mtb infection may contribute to the *in vivo* function restricting Mtb infection or dissemination while these expanded γδ T cells are actively mounting anti-TB Th1-like IFNγ cellular responses. The phosphoantigen/IL2-expanded Vγ2Vδ2 T cells appear to differentiate into multi-functional effector cell subpopulations that are able to mount anti-Mtb effector functions via producing at least IFNγ, perforin and granulysin. It is noteworthy that Vγ2Vδ2 T effector cells producing granulysin/perforin are able to limit Mtb growth in macrophages. The capability of Vγ2Vδ2 T effector cells to co-produce granulysin and perforin seems to be necessary for anti-Mtb effector function of Vγ2Vδ2 T effector cells as macaque granulysin, like human counterpart, exerts bactericidal effect on extracellular Mtb and inhibits intracellular Mtb in presence of perforin [35]. Earlier human studies showed that potent *in vitro* stimulation of human Vγ2Vδ2 T cells allowed these highly-activated γδ T cells to inhibit Mtb growth and that anti-perforin/anti-granulysin antibodies could reduce the anti-Mtb effector function [8], [32], [37], [39]. Now, the current study extended the previous *in vitro* work by demonstrating both the *in vivo* co-production of granulysin/perforin by Vγ2Vδ2 T effector cells in lung tissues and the direct anti-Mtb activities exerted by these two cytokine proteins. Our findings support the notion that anti-Mtb Vγ2Vδ2 T effector cells and granulysin/perforin effector cytokines might contribute to phosphoantigen/IL2-induced resistance to Mtb infection or TB lesions, although down-regulation of Treg for enhancing immune responses might also play a role. Thus, phosphoantigen-differentiated Vγ2Vδ2 T effector cells and *in vivo* co-production of granulysin/perforin in lungs may be relevant to a mechanism underlying the *in vivo* control of Mtb replication or dissemination in Picostim/IL2-treated macaques.

Another surprising observation is that Picostim/IL2-expanded Vγ2Vδ2 T cells are able to constitutively produce IL-12 without Ag stimulation, and expansion of Vγ2Vδ2 T cells leads to enhanced pulmonary immune responses of Mtb-specific αβ T effector cells. IL-12, a major cytokine critical for eliciting Th1 immune responses, is usually produced by DC or other APC, but not T cells. Our demonstration of IL-12 production by Vγ2Vδ2 T effector cells is therefore considered novel. The constitutive production of IL-12 by Vγ2Vδ2 T effector cells helps to explain indirectly how expansion/differentiation of Vγ2Vδ2 T effector cells by phosphoantigen/IL2 could promote pulmonary Th1-like immune responses of CD4+ and CD8+ T cells in Mtb infection. In fact, frequencies of pulmonary CD4+ and CD8+ Th1-like cells in Picostim/IL2 group are significantly higher than those in saline/BSA or IL2 alone control during primary Mtb infection. The reason why enhanced responses were seen prominent only in pulmonary compartment but not in blood may be due to the fact that pulmonary Mtb infection leads to dominance of immune responses in lung but not in peripheral blood [40]. These results suggest that phosphoantigen expansion of Vγ2Vδ2 T effector cells may serve as immune adjuvant enhancing CD4+ and CD8+ Th1 responses. The finding also suggests that early expansion/differentiation of Vγ2Vδ2 T cells by phosphoantigen/IL2 during Mtb infection may not only limit Mtb replication and dissemination, but also help promote primary anti-TB CD4+ and CD8+ Th1 responses. The surprising *in vivo* observation of Vγ2Vδ2 T cell-driven anti-TB immunity would open an avenue for further mechanistic studies.

To our knowledge, the current study also represents the first appreciable attempt that amplification and differentiation of a potentially protective Ag-specific T-cell subpopulation in Mtb infection can confer detectable efficacy of resistance to TB in primates. Since non-human primates resemble their human counterparts in biology and host responses to TB, our findings may help to rationalize future studies for evaluating γδ T cell-targeted treatment modality in chronic TB. Notably, human Vγ2Vδ2 T cells can be selectively expanded in vivo by zoledronate, a clinical drug widely used for treatment of bone-related disease, and that zoledronate/IL-2 expansion of Vγ2Vδ2 T cells has been tested as therapeutic regimen for treatment of metastatic cancers [18], [19].

Thus, specific phosphoantigen/IL2 immune stimulation early during Mtb infection rapidly expands and differentiates Vγ2Vδ2 T cells into multi-functional effector cells capable of mounting anti-TB IFNγ response, inhibiting Mtb growth through *in vivo* or constitutive production of perforin/granulysin, and producing IL-12 and promoting pulmonary Th1 responses of peptide-specific CD4+ and CD8+ T cells. The observation that phosphoantigen/IL2 expansion/differentiation of Vγ2Vδ2 T cells increases resistance to TB in nonhuman primates provides a rationale to design further studies of treatment of established TB. Such future studies might help guide single or adjunctive phosphoantigen expansion/differentition of Vγ2Vδ2 T cells for treatment of MDR-TB or AIDS-related TB in humans.

## Materials and Methods

### Ethics statement

The use of macaques and experimental procedures were approved by Institutional Animal Care and Use Committee and Biosafety Committee (Protocols A 05-072 and A 08-029), University of Illinois College of Medicine at Chicago (UIC), and we followed the national and international guidelines [International Primatological Society (IPS) International Guidelines for the acquisition, care and breeding of nonhuman primates] regarding “The use of non-human primates in research” to minimize potential suffering of the studied macaques. Daily or weekly clinical follow-up were taken to ensure that animals were not suffering from severe coughing, respiratory distress, depression, refusing to take food, body-weight loss or other potential life-threatening signs. Humane euthanization procedures were immediately taken if those signs occurred progressively.

### Animals

Twenty-seven cynomolgus macaques (*M. fascicularis*), 4–8 year old and 3–5 kg, were used in this study. All animals were maintained and used in accordance with guidelines of the institutional animal care and use committee(IACUC). Animals were anesthetized with 10 mg/kg ketamine HCl (Fort Dodge Animal Health, Fort Dodge, IA) i.m. for infection, bronchoalveolar lavage(BAL), blood samplings and treatments. All experimental protocols were approved by IACUC and IBC in University of Illinois Chicago.

#### Mtb infection

500 CFU Mtb Erdman were spread into bronchoaleveolar interface of the right caudal lung lobe using bronchoscope-guided challenge technique as we previously described [21], [27], [29]. This dose and approach consistently induced severe TB in a total of 21 naive macaques including 9 in this study that we had tested [27], [29], [41], [42], [43].

### Picostim and IL-2 administration, and controls

Picostim and HMBPP phosphoantigen chemicals are very similar in bioactivity and structure as they differ in only one atom linking the carbon chain and the phosphate moiety [15]. Both Picostim and HMBPP are specifically recognized by TCR expressed on primate Vγ2Vδ2 T cells [13] not by other γδ T cell subsets or αβ T cells. Picostim (>98 pure) was synthesized and provided by the company Innate Pharma(France). In the Picostim/IL2 co-treated group(n = 9), Picostim (25 mg/kg) was administered i.m., as we previously reported [15], on day −3 and day 15, respectively, after Mtb infection; recombinant human IL-2 (rhIL-2; Proleukin, Chiron, Emeryville, CA) was given i.m. at a dose of 1.2 million IU IL-2 once daily for 5 consecutive days beginning on the day of Picostim treatment. As a control group(n = 9), 1.2 million IU IL-2/day was given once daily i.m. for 5 consecutive days. As another control(n = 9), five animals received saline and four received bovine serum albumin (BSA, 27.2 ug) in 0.5-ml s.c. for 5 consecutive days. All treated macaques did not exhibit any notable side effects during treatments.

#### Bronchoalveolar lavage (BAL)

This was done as we previously described [9], [21], [27], [29]. Briefly, prior to BAL, animals were subjected to overnight fasting, and were tranquilized i.m. with 1–2 mg/kg xylazine (Ben Venue Laboratories, Bedford, OH) and 10 mg/kg ketamine HCl. For BAL, animals also received 0.05 mg/kg atropine (Phoenix Scientific, Inc., St. Joseph, MO) i.m. as an anticholinergic and were restrained in an upright position. A pediatric feeding tube was inserted down into the trachea through direct visualization with a laryngoscope and further into the right or left bronchus at the level of the carina. 10 ml of saline were instilled into the bronchus and immediately withdrawn and repeated a maximum of 3 times until a total of 12–15 ml BAL fluid was retrieved. This procedure generally gave rise to fluid compositions and volumes that were comparable to the bronchoscope-guided BAL (data not shown).

#### Isolation of single cell suspensions and lymphocytes from blood and BAL fluid

These methods were described in details in our recent publications [21], [27], [29].

#### Antigens (Ag) and Antibodies (Ab)

The phosphoantigen compound, nonpeptidic phosphorylated metabolite of isoprenoid biosynthesis, [e.g. (*E*)-4-hydroxy-3-methyl-but-2-enyl pyrophosphate (HMBPP)] specifically recognized by primate Vγ2Vδ2 T cells not by others, was produced, characterized, validated and provided by Dr. Hassan Jomaa in Giessen, Germany. The purity of HMBPP was >98% [16]. The phosphoantigen compound Picostim shares with HMBPP the capacity to specifically activate and expand Vγ2Vδ2 T cells[see above and [15]]. PPD were purchased from Mycos Research (Loveland, CO). Anti-CD28 (CD28.2, BD) and anti CD49d (9F10, BD) were used in the assays as co-stimulatory Abs. The following mAbs were used for surface and intracellular cytokine staining for flow cytometry analyses: anti-human CD3 (SP34-2, BD), anti-human CD4 (OKT4, eBioscience), anti-human CD8 (DK25, Dako), anti-human Vγ2 (7A5, Thermo), anti-human Vδ2(15D, Pierce, IL), anti-human Vδ1(TS8, Pierce), anti-human Vδ3(P11.B, Pierce), anti-human IFNγ-Allophycocyanin (4S.B3, BD), Perforin-biotin (Pf-344, Mabtech, Cincinnati, OH), anti-human granulysin-PE (clone DH2, Novus Biologicals), Anti-human IL-12-PE (clone C8.6, Miltenyi), Isotype control Abs: purified mouse IgG isotype or mouse IgG-PE (eBioscience).

#### Immunofluorescent staining and flow cytometric analysis

These were described in details in our recent publications [15], [17], [27]. Briefly, PBMC and BAL cells were stained with up to 5 Abs (conjugated to FITC, PE, allophycocyanin, pacific blue, and PE-Cy5 or allophycocyanin-Cy7) for at least 15 min(Staining panels: CD3/CD4/CD8/Vγ2/Vδ2;CD3/pan-γδ/Vδ1 or Vδ3) After staining, cells were fixed with 2% formaldehyde-PBS (Protocol Formalin, Kalamazoo, MI) prior to analysis on a CyAn ADP flow cytometer (DakoCytomation, Carpinteria, CA). Lymphocytes were gated based on forward- and side-scatters, and pulse-width and at least 40,000 gated events were analyzed using Summit Data Acquisition and Analysis Software (DakoCytomation). Absolute cell numbers were calculated based on flow cytometry data and complete blood counts performed on a hematology system (Advia 120, Siemens, Tarrytown, NY).

#### Intracellular cytokine staining (ICS) for detection of T effector cells

ICS was done as previously described [21], [27], [29]. 10^5^–10^6^ BAL cells or 10^6^ PBMC plus mAbs CD28 (1 µg/ml) and CD49d (1 µg/ml) were incubated with PPD (25 ug/ml, from Mycos Research (Loveland, CO), HMBPP(80 ng/ml) or media alone in 100 µl final volume for 1 h at 37°C, 5% CO_2_ followed by an additional 5 h incubation in the presence of brefeldin A (GolgiPlug, BD). After staining cell-surface CD3, CD4, CD8 or Vγ2 for 30 min, cells were permeabilized for 45 min (Cytofix/cytoperm, BD) and stained for another 45 min to detect IFNγ, perforin or IL-12 before re-suspending in 2% formaldehyde-PBS. ICS panels: CD3-PEcy7/CD4-APC/Vγ2-FITC/IFNγ-PE/perforin-PB;CD3-PEcy7/CD8-APC/Vγ2-FITC/IFNγ-PE/perforin-PB;CD3-PEcy7/CD4-APC/Vγ2-FITC/IL12-PE; CD3-PEcy7/CD4-APC/Vγ2-FITC/mouse IgG-PE. No positive staining of PBMC or BAL samples collected overtime after infection/treatments was seen in ICS when using the mouse IgG isotype control(data not shown).

#### Direct ICS for measuring T effector cells constitutive producing cytokines without in vitro antigen stimulation

Direct ICS was adapted from the conventional ICS protocol, and the major modification was that PBMC or BAL cells were directly measured by ICS for intracellular cytokines without prior *in vitro* Ag stimulation as we recently described [17], [27], [28], [29], [44]. Direct ICS approach was characterized and validated extensively *in vitro* and *in vivo* conditions as we previously reported [17], [22], [27], [28], [29], [30]. Briefly, PE-mouse IgG served as internal isotype control and did not yield any positive ICS staining over time after treatments (data not shown). As *in vivo* controls, longitudinal direct ICS analyses of PBMC or BAL cells obtained biweekly for 6 weeks from 3 normal uninfected macaques or during acute- and chronic-SHIV-infection of four macaques did not detect measurable increases in IFNγ- or perforin-producing Vγ2 or CD4+ T cells in PBMC [17], [28]. In addition, direct ICS did not detect increased levels of IL-17- or IL-22-producing T-cells in blood over time during acute- and chronic-SHIV-infection [17], [27].

#### *In situ* confocal microscopic analysis of Vγ2 TCR, perforin and granulysin in lung tissue sections of Mtb-infected macaques

The OCT lung tissue blocks were cut into 6 um sections using a cryostat as we previously described [14], [29], [42]. The frozen tissue sections were fixed with acetone for 10 min, rinsed with PBS and then blocked with Protein Blocking Serum-Free buffer (Dako, X0909) for 20 min. Sections were incubated for 1 hr in dark with FITC-conjugated anti-human TCR Vγ2 mAb (clone 7A5, Thermo), 1∶10 diluted biotinylated anti-human perforin mAb (clone pf-344, MABTECH AB) and PE-conjugated anti-human granulysin mAb (clone DH2, Novus Biologicals). The sections were rinsed with PBS, and then incubated for 30 min in dark with APC-conjugated streptavidin (Cat# 405207, Biolegend). The sections were then rinsed with 0.02% Tween20-PBS, and cover-slipped and mounted on slides using fluorescence mounting medium with 4′, 6-diamidino-2-phenylindole (DAPI) (H-1200, Vector). The edges of cover slip were sealed with nail polish. The slides were imaged by a Carl Zeiss (Thornwood, NY) LSM510 Meta5 laser scanning confocal microscope equipped with a 63×/1.2 water-immersion objective. FITC-conjugated mouse IgG served as isotype control staining, and did not give rise to fluorescent images on the sections under the confocal(data not shown).

#### Bacterial colony forming units (CFU) counts in lung lobe tissue homogenates

To objectively measure CFU counts in lung lobes, we used the sampling strategy and CFU determination as previously described [21], [22], [27], [29], [45]. Briefly, approximately a half of cut-sections of the right caudal lobe (the infection site), the right middle lobe or the left caudal lobe from each animal were taken for CFU determination after the extensive gross pathologic evaluation was accomplished. If there were gross TB lesions in the respective lobe, a half of the lung tissue containing approximately 50% lesions was taken. If no visible lesions were seen in the respective lobe, a random half of tissue was taken for evaluation. Tissue homogenates were made using a homogenizer (PRO 200, PRO Scientific INC, CT) and diluting the homogenate in sterile PBS+0.05% Tween-80. 5-fold serial dilutions of samples were plated on Middlebrook 7H11 plate(BD).

#### Gross pathologic analysis and the scoring system measuring TB lesions

Details were described in our previous publications [21], [22]. Briefly, animals were euthanized by intravenous barbiturate overdose, and immediately necropsied in a biological safety cabinet within a BSL-3 facility. Standard gross pathologic evaluation procedures were followed by the blinded pathologist and associates, with each step recorded and photographed at day 65 after Mtb infection. Lung lobes, bronchial, mesenteric, axillary and inguinal lymph nodes, tonsils, and other major organs were collected and labeled. Multiple specimens from all tissues with gross lesions and remaining major organs were harvested. Gross observations including but not limited to the presence, location, size, number and distribution of lesions were recorded. The scoring system [20], [21], [22] was excised to calculate gross pathology scores for TB lesions in lungs infected by bronchoscope-guided inoculation. For each of lung lobes, granuloma prevalence was scored 0–4 for (i) no visible granulomas, (ii) 1–3 visible granulomas, (iii) 4–10 visible granulomas, (iv) >10 visible granulomas, and (v) miliary pattern of granulomas, respectively. Granuloma size was scored 0–3 for (i) none present, (ii) <1–2 mm, (iii) 3–4 mm, and (iv) >4 mm, respectively. Pulmonary consolidation or atelectasis as viewed from organ exterior and cut surfaces were scored 0–2 for (i) absent, (ii) present focally in one lobe, and (iii) focally extensive within a lobe or involving multiple lobes. One score was also given for the presence of tuberculosis-related focal parietal pleural adhesions, pleural thickening and opacification, or pulmonary parenchymal cavitation. For hilar lymph nodes, enlargements were scored 0–3 for (i) visible but not enlarged, (ii) visibly enlarged unilaterally (≤2 cm), (iii) visibly enlarged bilaterally (≤2 cm), (iv) visibly enlarged unilaterally or bilaterally >2 cm, respectively. Tuberculosis lesions in hilar lymph nodes were scored 0–4 for (i) no granulomas visible on capsular or cut surface, (ii) focal or multifocal, circumscribed, non-coalescing granulomas, <2 mm, (iii) coalescing solid or caseating granulomas occupying<50% of nodal architecture, (iv) coalescing solid or caseating granulomas occupying >50% of nodal architecture, with residual nodal components still recognizable, and (v) complete granulomatous nodal effacement and caseation, respectively. One score was also given for tuberculosis-associated changes in other thoracic nodes. The tuberculosis lesions in each of extrathoracic organs were scored similarly as each lung lobe. The pathology scoring of infected tissues was conducted in a blinded fashion.

#### Microscopic analysis of TB lesions

This was done as we previously described [21], [41], [44]. Briefly, the extent of involvement for each lung lobe was determined using digital scans to record total pixel counts on H&E stained material and specimen area measured in square cm using Image-Pro Plus software (MediaCybernetics, Silver Spring, MD), as previously described [21], [22], [41], [45]. Granulomas were objectively compared for size, type, distribution pattern and cellular composition (absence or presence with degrees of lymphocytic cuff, mineralization, fibrosis, multinucleated giant cells, and epithelioid macrophages) between and within monkey groups.

#### Vγ2Vδ2 T effector cell-based inhibition of intracellular Mtb growth

This was done through co-culturing Mtb-infected autologous monocytes/macrophages and Vγ2Vδ2 T effector cell lines as we reported [29]. Phosphoantigen-activated Vγ2Vδ2 T effector cell lines were generated from three Mtb-exposed macaques using the approach as previously described [21]. Briefly, Vδ2 T cells were purified by immunomagnetic beads [15], and then activated and expanded through co-culturing with BCG-infected autologous monocyte-derived DC [29] in the conditioned growth medium as previously described [31]. Up to 90% of these Vδ2+ T cells co-expressed Vγ2(data not shown) after the 14-day co-culture with BCG-infected DC in the conditioned medium [31]. To generate Mtb-infected monocytes/macrophages, PBMC were cultured on plastic tissue culture dishes for 4 days at 37°C, 5% CO_2_, thereafter non-adherent cells were discarded, and adherent cells were collected by scraping with a cell scraper. The cell suspension contained 80–90% macrophages. Adherent monolayers (5×10^4^/well in 96-well plate) were infected with Mtb at an MOI of 10∶1. After 3 h of incubation at 37°C, supernatants were aspirated and wells were washed three times to remove non-ingested mycobacteria. Mtb-infected monocytes were then co-cultured at a ratio of 1∶10 with Vγ2Vδ2 T effector cell lines (5×10^5^/well) in multiple wells of a 96-well plate. Bead-enriched autologous CD20^+^ B cells served as a control. After co-culturing for 72 hrs, cells were lysed in lysis buffer (0.067% SDS in Middlebrook 7H9), and lysates were plated in 10-fold serial dilutions on Middlebrook 7H11 agar(BD) and incubated for 3 weeks in 5% CO_2_ at 37°C.

#### Production of soluble macaque granulysin and perforin proteins

Macaque full-length granulysin and perforin cDNA clones [41] were used to subclone DNA constructs that encode the active granulysin bearing C-terminal portion 83 amino acid (aa) residues and the active perforin comprised of C-terminal portion 125 aa, respectively, using the PCR-based cloning approach [34]. The purified PCR products were digested, ligated to the vector *pET-30* (Novagen) and transformed into *E. coli* competent cells *BL21* (DE3, Novagen). Selected cells with correct constructs were cultured in LB- kanamycin (30 µg/ml) medium overnight, and once the cultured medium reached about 0.6 at OD_600_, 1 mmol/L of *isopropyl thio-β-D-galactoside* (IPTG) was added to induce protein expression via vigorous shaking. The expression cells were pelleted and lysed to collect the cytoplasm and inclusion bodies, and characterized for expression and location of these proteins by SDS-PAGE and Western blot assays. To purify recombinant proteins, the cell pellets were harvested from large-scale expression (induced for 5∼12 hours at 28°C) by centrifugation, resuspended for 30 minutes in 20 mM/L of Tris-HCl (pH 7.6) buffer containing 20 mg/ml of lysozyme, and then lysed by sonication (Microson) for 5×10 seconds in ice-bath. The lysates were centrifuged at 10,000×g for 30 minutes at 4°C to remove the supernatants, and the collected inclusion bodies containing recombinant proteins were solubilized with Guanidinium Buffer (Guanidine HCl 6M/L, NaCl 500 mM/L, sodium phosphate 20 mM/L, PMSF 0.1 mM/L). Soluble samples were bound to Ni-NTA agarose (Qiagen) to purify 6×His tagged granulysin or perforin by affinity chromatography under denaturing conditions. Redox environment, buffer composition, protein concentration and temperature of refolding procedures were examined to optimize the refolding conditions for the recombinant proteins using Pro-Matrix protein refolding kit (Pierce). A presence of detection tags (S- and His-tag) in the target protein was monitored for the success of refolding by SDS-PAGE and Western blot. Refolded samples were then passed through a S-protein agarose (Novagen) column to purify S-tagged proteins. After dialysis with PBS and concentration by 10∼20 K cut-off iCON concentrator (Pierce), recombinant Enterokinase (rEK, Novagen) was used to cleave the specific cleavage site of AspAspAspAspLys between S-tag sequence and expressed target protein. After removing the free rEK and the N-terminal S-tag portion by Enterokinase Cleavage Capture Kit (Novagen), purified recombinant protein was evaluated for its molecular mass weight by SDS-PAGE analysis and measured for concentration using BCA protein assay kit (Pierce). Specific anti-human granulysin C domain [37] and perforin C-terminus antibodies [46], [47] were used in Western blot assays, as previously described [13], [34], to confirm purified granulysin and perforin.

#### Macaque granulysin-mediated killing of extracellular Mtb

5×10^3^ CFU of Mtb H37Rv suspension were distributed in each well of a 96-well plate in presence of 50 µM, 10 µM, 1.0 µM, or 0.1 µM (triplicate for each) purified granulysin or perforin protein at a final volume of 50 µl 7H9 medium, and then incubated in 5% CO_2_ at 37°C for 72 hours. As negative controls, medium only and four different concentrations of recombinant heat shock protein (HSP70, expressed in *E. coli*, Sigma) were similarly added to individual wells with defined CFU of Mtb. The samples were then processed in ten-fold serial dilutions with Middlebrook 7H9 broth, and each dilution was plated for CFU on Middlebrook 7H11 agar(BD) and incubated in 5% CO_2_ at 37°C 3–4 weeks. The killing percentage of extracellular Mtb bacteria was calculated as [CFUs in medium-only control - CFUs in granulysin or perforin or HSP70]/CFUs in medium-only control [37].

#### Macaque granulysin/perforin-mediated inhibition of intracellular Mtb

Monocytes as prepared above were infected with Mtb H37Rv using the similar procedure as described above. Mtb-infected monocytes were distributed in a 96-well plate at a density of 1.0×10^4^ per well in presence of 0.1 µM, 1.0 µM, 10 µM and 50 µM(duplicate for each concentration) of recombinant granulysin or perforin or both. The co-cultures were incubated in 5% CO_2_ at 37°C in a final volume of 100 µl per well with RPIM-1640 medium. HSP 70 and medium-only treatments were included as negative controls, respectively. After 72 hours culture, the cells were lysed, and the lysates were measured for CFU in 10-fold serial dilutions as described above. The inhibiting percentage of intracellular Mtb bacteria was calculated as [CFUs in monocytes treated with medium-only control - CFUs in monocytes treated with granulysin, perforin or HSP70]/CFUs in monocytes treated with medium-only control [37].

#### Statistical analysis

The multivariate analysis of variance (ANOVA) and student *t* test were used, as previously described [14], [21], to statistically analyze the data for differences between picostim/IL2-treated and control groups. Wherever non-gaussian distribution of data is occurring/expected, e.g. with scoring of pathology, non-parametric analysis was utilized. When non parametric statistical analysis of CFU and pathology scores was performed, similar results were seen as the above parametric method(data not shown). Since limited numbers of macaques/group were employed in this proof-of-concept study, medians, not means, of CFU and gross pathology scores were shown in the respective figures and used for statistical analyses(significance is similar as the means).

## Supporting Information

Text S1 **Fig. S1.** Picostim/IL2 treatment, while expanding Vγ2Vδ2 T cells, could confer immune resistance to TB lesions in lungs after pulmonary Mtb infection. Shown are dditional digital photos of cut sections of lung lobes from other macaques(a total of 27). Please see Fig. 2 legend in Text for detailed description of photos. **Fig. S2.** Shown are additional histopathology photos for other macaques from Picostim/IL-2-treated and control groups. Please see Fig. 3b legend in Text for detailed description of photos. Original magnification ×100 for all photos. **Fig. S3a.** Phosphoantigen-expanded Vγ2Vδ2 T cells in pulmonary compartments possess the capacity to *de novo* produce anti-Mtb cytokines IFNγ, perforin and granulysin without phosphoantigen HMBPP stimulation in vitro. Shown are representative flow cytometry histograms (left) of IFNγ-producing(top panels) and perforin-producing(lower panels) Vγ2Vδ2 T effector cells gated on CD3 and graph data (right) of numbers of Vγ2Vδ2 T effector cells in BALF collected overtime from Picostim/IL-2-treated and control groups. Effector cells were measured by ICS without HMBPP stimulation. See Fig. 4a legend in Text for detailed description. **Fig. S3b.** Additional representative *in situ* confocal microscopic images (63× NA) of Vγ2Vδ2 T effector cells producing perforin and granulysin in lung tissue sections from other macaques. See Fig. 4b legend in Text for detailed description. **Fig. S3c.** Immunohistochemistry analysis of Vγ2 T cells in lung parenchyma and granuloma tissues. Note that more Vγ2 T cells were detected in “tiny”, small and large granulomas tissues in Picostim/IL2-treated macaques than those in control IL2 alone- and saline/BSA-treated macaques. Magnifications were indicated. Immunohistochemistry analysis of Vγ2 T cells was essentially the same as previously described. **Fig. S3d.** Vγ2Vδ2 T effector cells that expanded and differentiated in vivo at day 14 after Picostim/IL-2 treatment could recognize Mtb-infected autologous macrophages, leading to inhibition of intracellular Mtb growth, and such inhibition could be reduced by antibodies against granulysin/perforin. Macaque PBMC frozen down at day 14 after Picostim/IL-2 treatment were cultured for 7 days in presence of HMBPP/IL2, and used to purify Vγ2Vδ2 T cells as described in Methods. Vγ2Vδ2 T cells were incubated for 4 days with autologous Mtb-infected monocytes(prepared using day 56 PBMC) at E∶T ratio of 10 in the presence of anti-perforin/granulysin Abs(see clones ID in Methods, 10 µg/ml for each) or IgG isotype control (10 ug/ml) as described in Methods. The cultured cells were lysed, and CFU counts in lysate were determined as described in Methods. N = 3. **Fig. S4.** Shown are SDS-PAGE and Western blot data for analysis of recombinant macaque perforin and granulaysin proteins purified from E-coli expression system [29]. See Fig. 5 legend in Text for details. **Fig. S5.** Picostim/IL-2 treated macaques exhibited greater numbers of IFNγ-producing CD4+ T cells (top) and CD8+ T cells(bottom) in BALF than saline/BSA-treated or IL-2-treated macaques. Effector cells were measured by direct ICS without antigen stimulation *in vitro*. See Fig. 6 legend in Text for details.
(PDF)
