# Supplementary material for: Phosphoantigen/IL2 Expansion and Differentiation of Vγ2Vδ2 T Cells Increase Resistance to Tuberculosis in Nonhuman Primates
Source: PLoS Pathog. 2013 Aug 15;9(8):e1003501. doi: 10.1371/journal.ppat.1003501 (PMC3744401; doi:10.1371/journal.ppat.1003501)
Supplement: Text S1 — Fig. S1. Picostim/IL2 treatment, while expanding Vγ2Vδ2 T cells, could confer immune resistance to TB lesions in lungs after pulmonary Mtb infection. Shown are dditional digital photos of cut sections of lung lobes from other macaques(a total of 27). Please see Fig. 2 legend in Text for detailed description of photos. Fig. S2. Shown are additional histopathology photos for other macaques from Picostim/IL-2-treated and control groups. Please see Fig. 3b legend in Text for detailed description of photos. Original magnification ×100 for all photos. Fig. S3a. Phosphoantigen-expanded Vγ2Vδ2 T cells in pulmonary compartments possess the capacity to de novo produce anti-Mtb cytokines IFNγ, perforin and granulysin without phosphoantigen HMBPP stimulation in vitro. Shown are representative flow cytometry histograms (left) of IFNγ-producing(top panels) and perforin-producing(lower panels) Vγ2Vδ2 T effector cells gated on CD3 and graph data (right) of numbers of Vγ2Vδ2 T effector cells in BALF collected overtime from Picostim/IL-2-treated and control groups. Effector cells were measured by ICS without HMBPP stimulation. See Fig. 4a legend in Text for detailed description. Fig. S3b. Additional representative in situ confocal microscopic images (63× NA) of Vγ2Vδ2 T effector cells producing perforin and granulysin in lung tissue sections from other macaques. See Fig. 4b legend in Text for detailed description. Fig. S3c. Immunohistochemistry analysis of Vγ2 T cells in lung parenchyma and granuloma tissues. Note that more Vγ2 T cells were detected in “tiny”, small and large granulomas tissues in Picostim/IL2-treated macaques than those in control IL2 alone- and saline/BSA-treated macaques. Magnifications were indicated. Immunohistochemistry analysis of Vγ2 T cells was essentially the same as previously described. Fig. S3d. Vγ2Vδ2 T effector cells that expanded and differentiated in vivo at day 14 after Picostim/IL-2 treatment could recognize Mtb-infected autologous macrophages, lea [file ppat.1003501.s001.pdf]

Supplementary Figures

Fig.S1

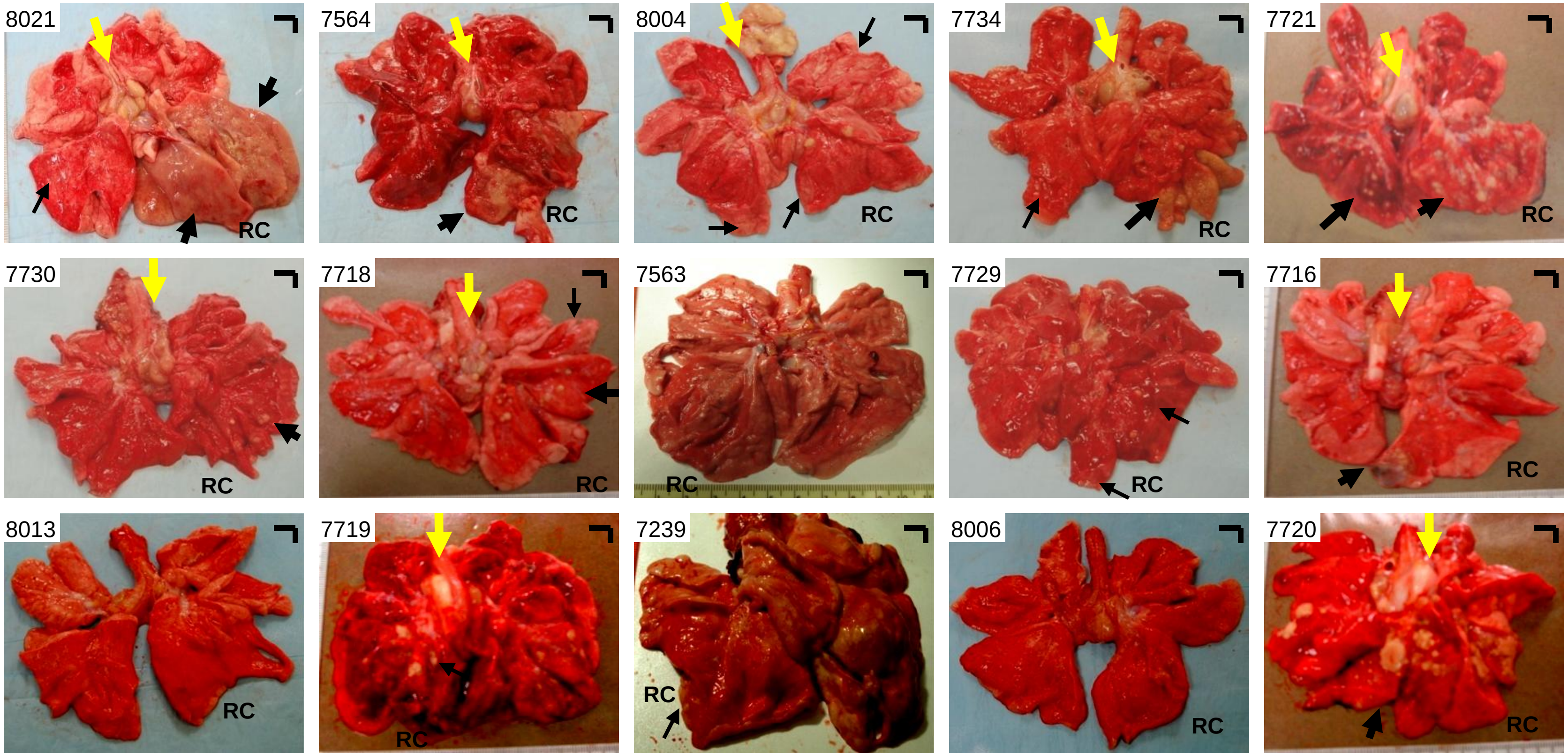

Fig.S2

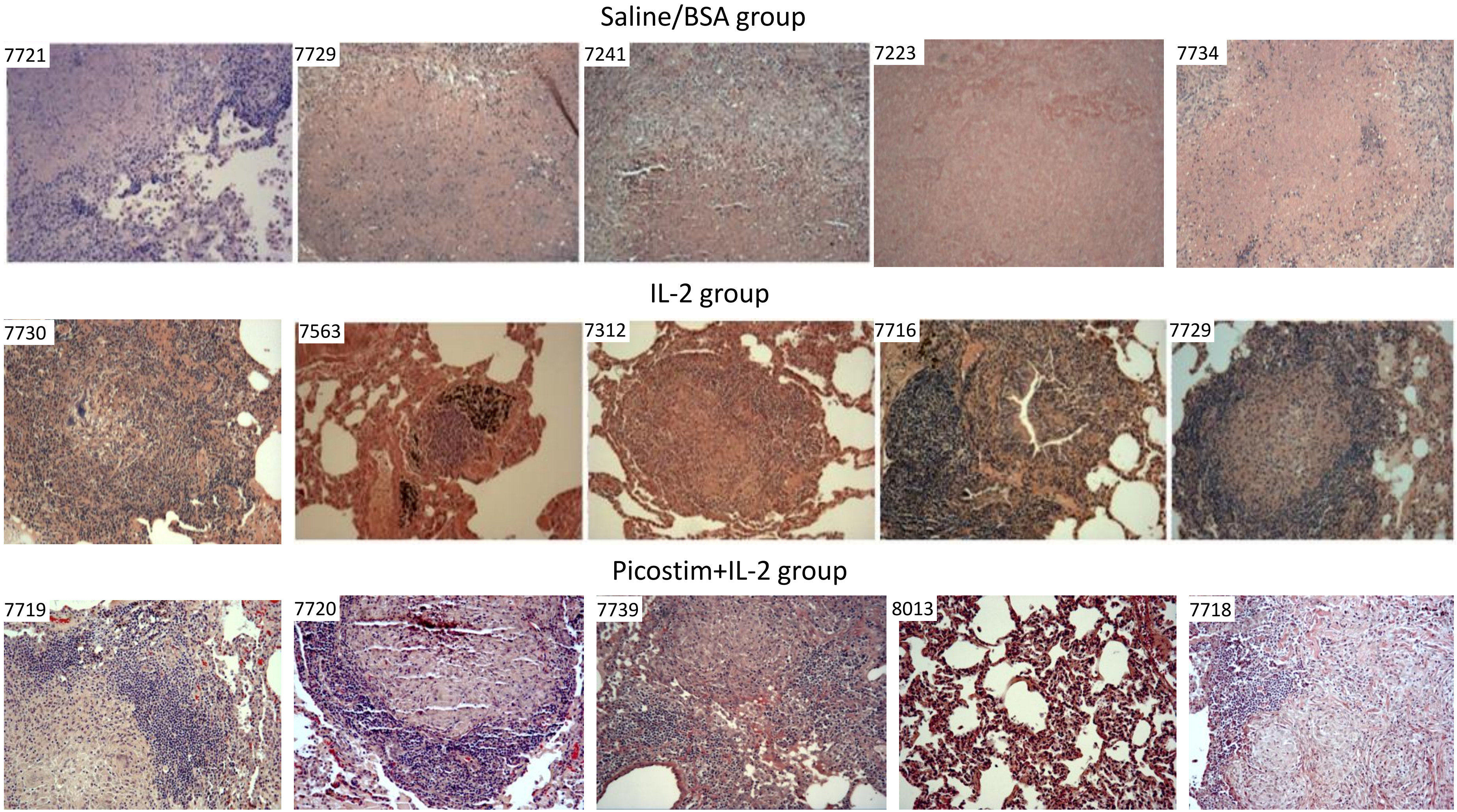
Saline/BSA group
7721
7729
7241
7223
7734
IL-2 group
7730
7563
7312
7716
7729
Picostim+IL-2 group
7719
7720
7739
8013
7718

Fig.S3a

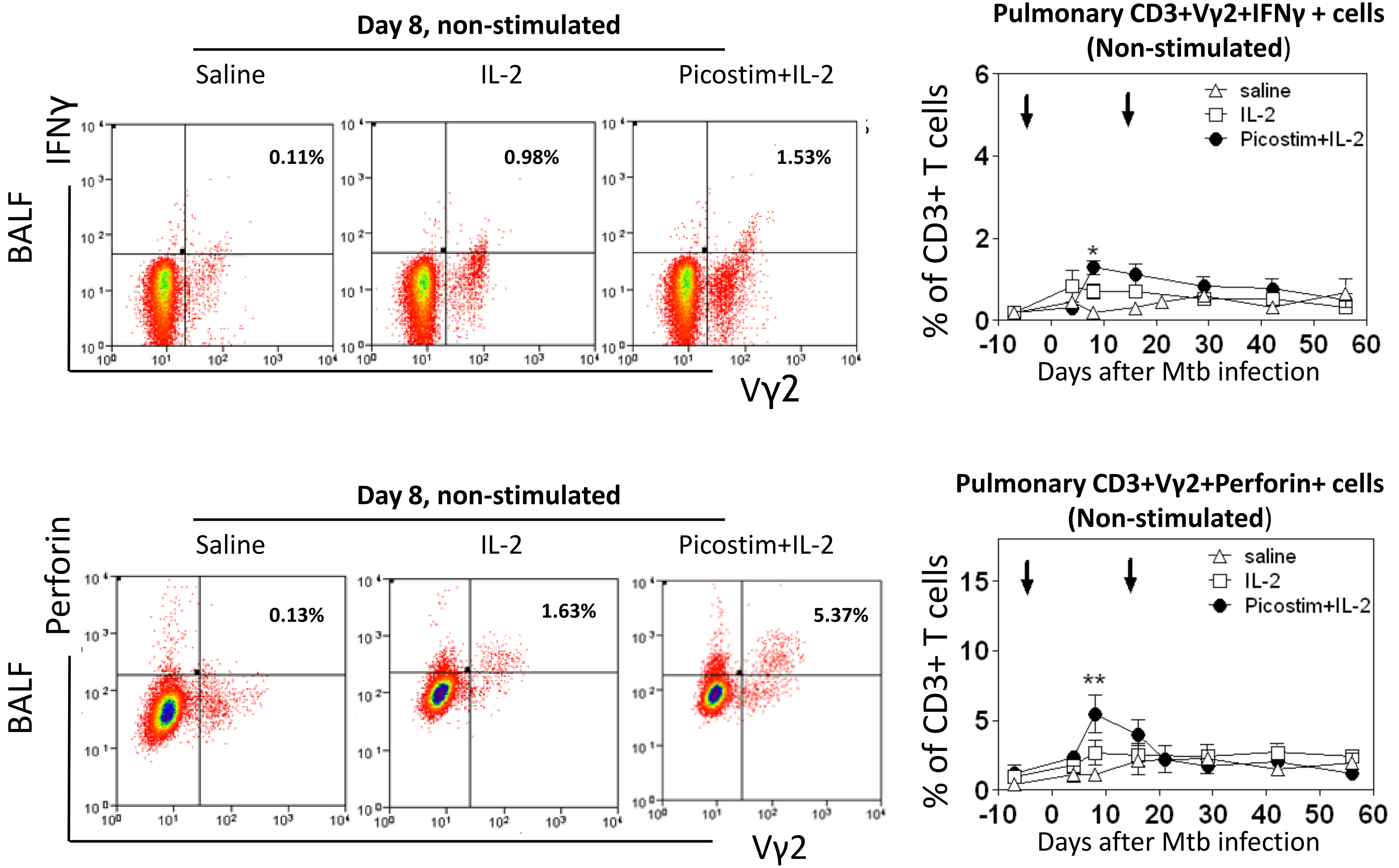

Day 8, non-stimulated
Saline
IL-2
Picostim+IL-2
BALF
IFNγ
0.11%
0.98%
1.53%
Vγ2
Pulmonary CD3+Vγ2+IFNγ + cells
(Non-stimulated)
% of CD3+ T cells
saline
IL-2
Picostim+IL-2
Days after Mtb infection
Day 8, non-stimulated
Saline
IL-2
Picostim+IL-2
BALF
Perforin
0.13%
1.63%
5.37%
Vγ2
Pulmonary CD3+Vγ2+Perforin+ cells
(Non-stimulated)
% of CD3+ T cells
saline
IL-2
Picostim+IL-2
Days after Mtb infection

Fig.S3b

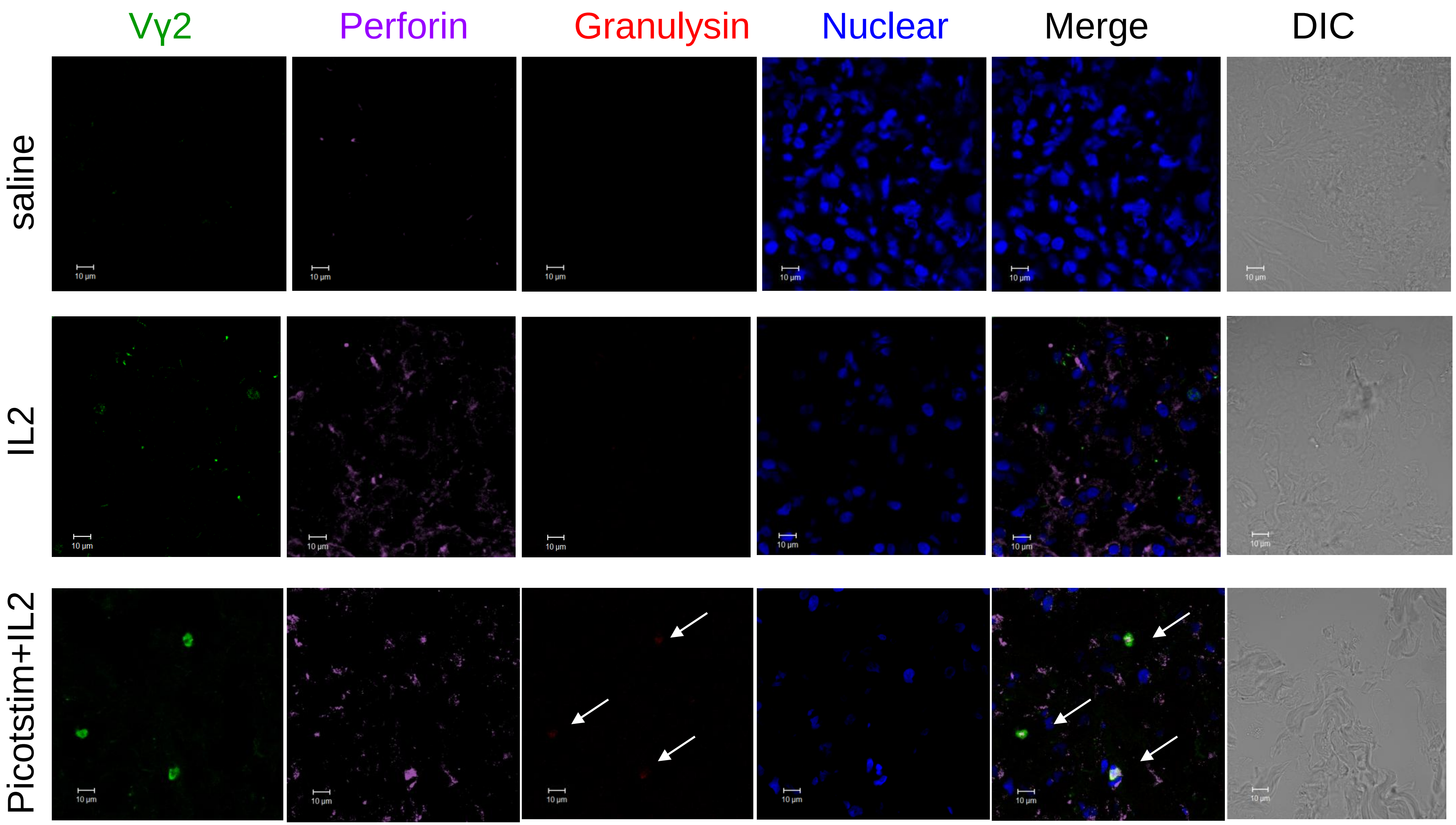
Vγ2
Perforin
Granulysin
Nuclear
Merge
DIC
saline
IL2
Picotstim+IL2
10 µm

Fig.S3c

Picostim + IL2

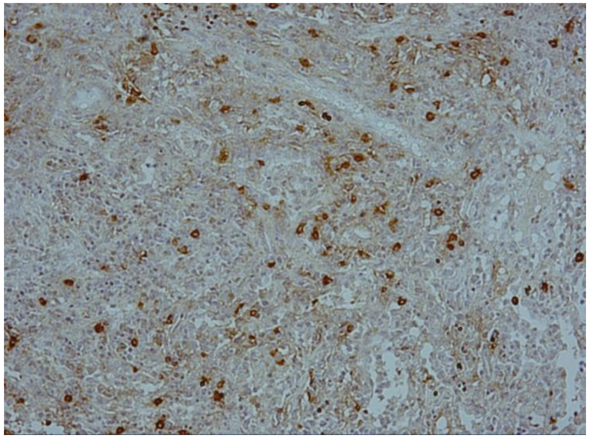

CN 7218: Many Vγ2 T cells seen in a large granuloma. x100

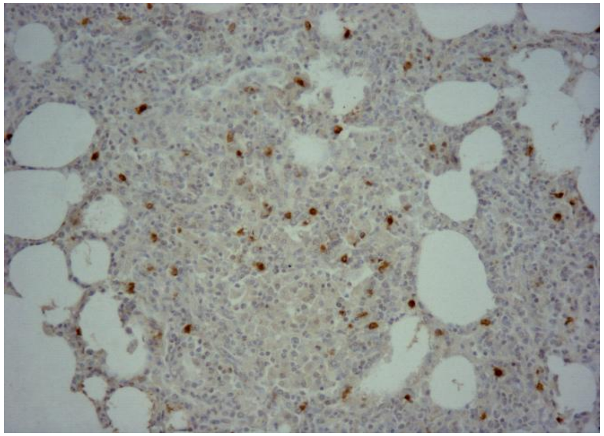

CN 8006 : Many Vγ2 T cells seen in a small granuloma. x100

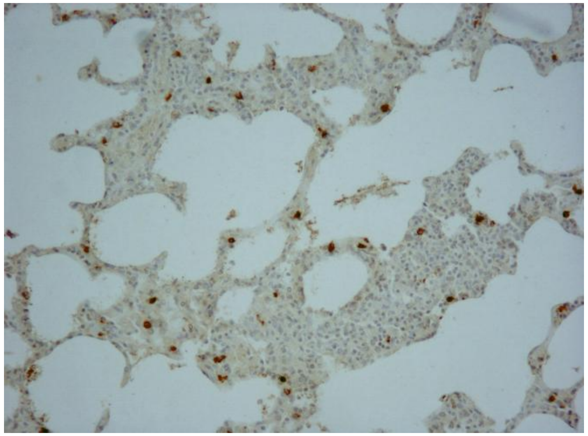

CN 7719: Many Vγ2 T cells seen in a tiny granuloma. x100

IL2

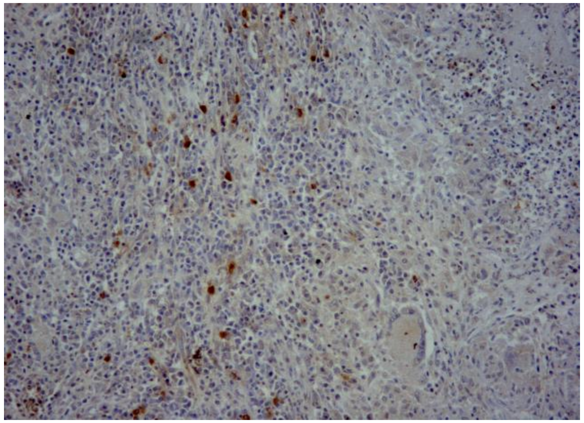

CN 7717: Some Vγ2 T cells seen in a large granuloma. x100

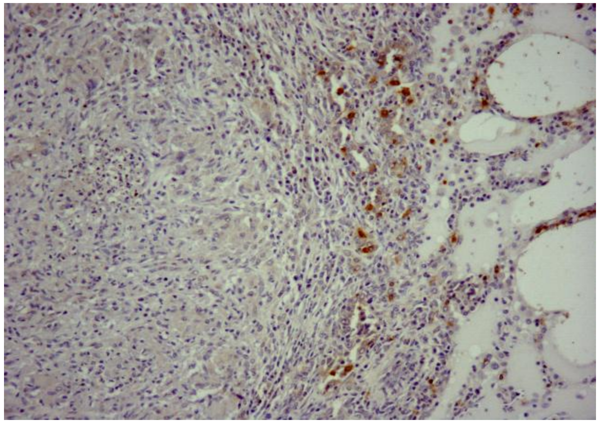

CN 8018: Some Vγ2 T cells seen around a small granuloma. x 100

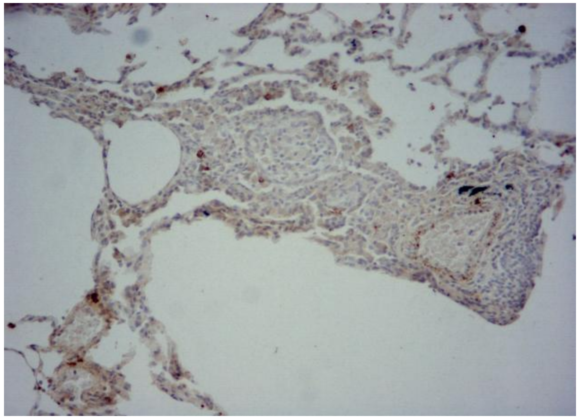

CN 8018 : Only 4 Vγ2 T cells seen in a tiny granuloma. x100

Saline

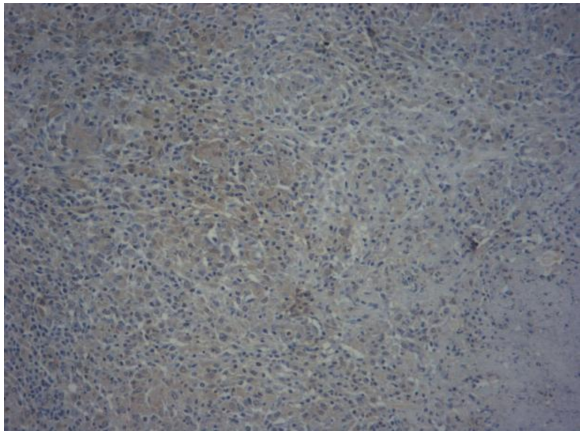

CN 7241: No Vγ2 T cells seen in the center of a large granuloma. x100

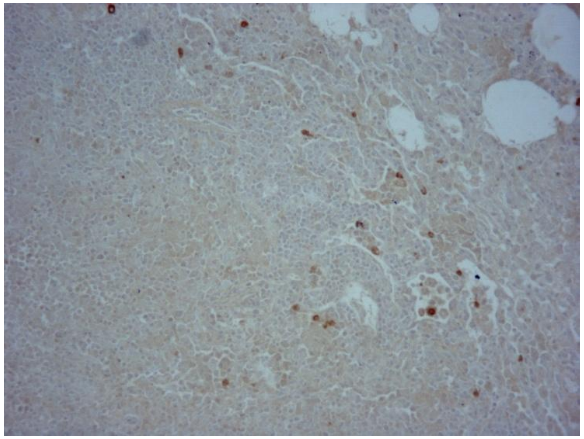

CN 7721: A few Vγ2 T cells seen in a tiny granuloma. x100

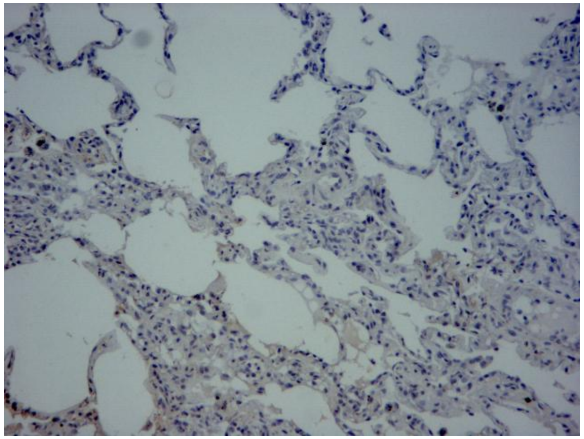

CN 7734: No Vγ2 T cells seen in a tiny granuloma. x100

Fig.S3d

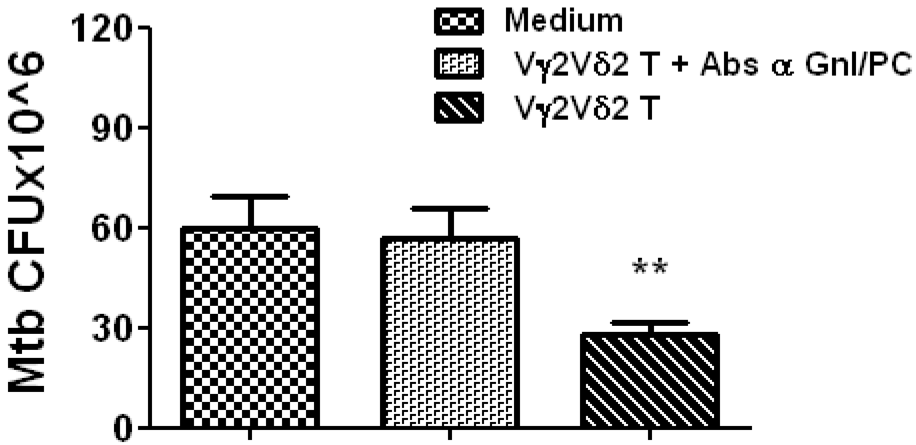

Fig.S4

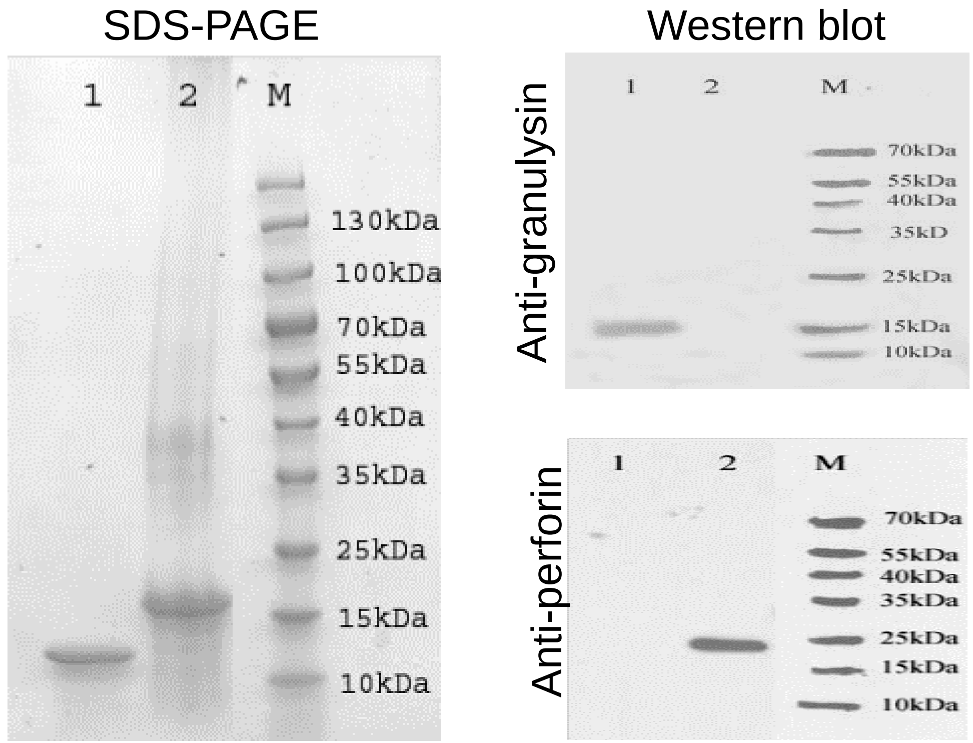
SDS-PAGE
1
2
M
130kDa
100kDa
70kDa
55kDa
40kDa
35kDa
25kDa
15kDa
10kDa
Western blot
Anti-granulysin
1
2
M
70kDa
55kDa
40kDa
35kD
25kDa
15kDa
10kDa
Anti-perforin
1
2
M
70kDa
55kDa
40kDa
35kDa
25kDa
15kDa
10kDa

Fig.S5

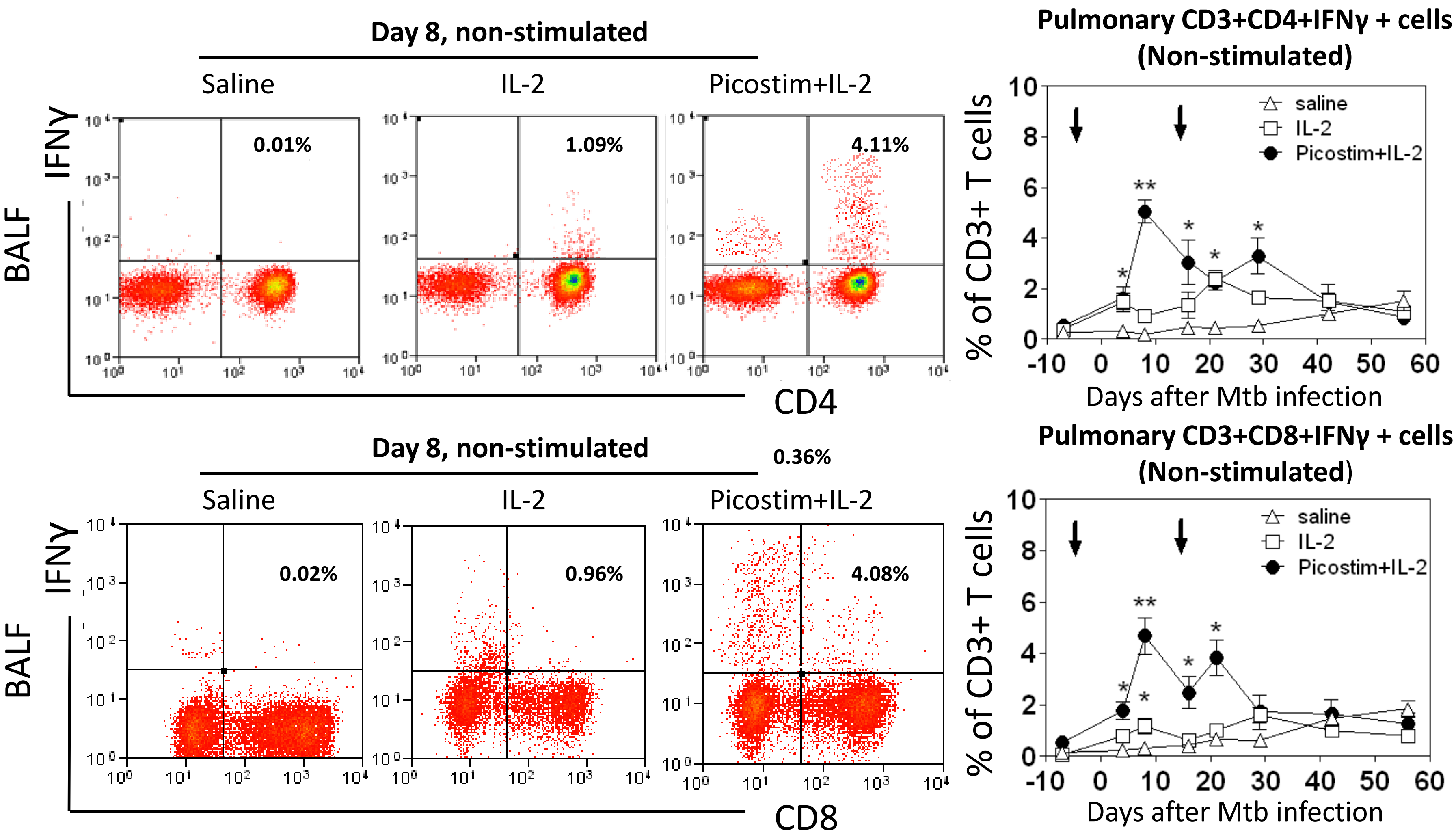
Day 8, non-stimulated
Saline
IL-2
Picostim+IL-2
IFNγ
BALF
0.01%
1.09%
4.11%
CD4
Pulmonary CD3+CD4+IFNγ + cells
(Non-stimulated)
% of CD3+ T cells
saline
IL-2
Picostim+IL-2
Days after Mtb infection
Day 8, non-stimulated
0.36%
Saline
IL-2
Picostim+IL-2
IFNγ
BALF
0.02%
0.96%
4.08%
CD8
Pulmonary CD3+CD8+IFNγ + cells
(Non-stimulated)
% of CD3+ T cells
saline
IL-2
Picostim+IL-2
Days after Mtb infection
